# A Platform for the Actuation of Magnetically Labeled Skeletal Muscle Cells Using Dynamic Magnetic Stimulation

**DOI:** 10.1002/smll.74473

**Published:** 2026-07-14

**Authors:** Tayná C. Rodrigues, Anna‐Lena Bauknecht, Anna Gioran, Sabine Rosenfeldt, Daniel Dickes, Johann Schorzmann, Frank Döpper, Elisabetta A. Cavalcanti‐Adam, Michael Maier, Reinhard Richter, Marissa K. Caldow, Gordon S. Lynch, Daniel E. Heath, Andrea J. O'Connor, Sahar Salehi

**Affiliations:** ^1^ Chair of Biomaterials Faculty of Engineering Science University of Bayreuth Bayreuth Germany; ^2^ Fraunhofer Institute for Manufacturing Engineering and Automation (IPA) Stuttgart Germany; ^3^ Department of Biointelligent System Engineering Institute of Food Science and Biotechnology University of Hohenheim Stuttgart Germany; ^4^ Department of Biomedical Engineering Graeme Clark Institute University of Melbourne Victoria Australia; ^5^ Empa Swiss Federal Laboratories for Materials Science and Technology Laboratory for Biointerfaces St. Gallen Switzerland; ^6^ Empa Swiss Federal Laboratories For Materials Science and Technology Laboratory for Biomimetic Membranes and Textiles St. Gallen Switzerland; ^7^ Chair of Physical Chemistry I Faculty of Biology Chemistry & Earth Sciences and Bavarian Polymer Institute University of Bayreuth Bayreuth Germany; ^8^ Chair for Metals and Alloys Faculty of Engineering Science University of Bayreuth Bayreuth Germany; ^9^ Chair for Manufacturing and Remanufacturing Technology Faculty of Engineering Science University of Bayreuth Bayreuth Germany; ^10^ Chair of Cellular Biomechanics Faculty of Engineering Science University of Bayreuth Bayreuth Germany; ^11^ Experimental Physics V Department of Physics University of Bayreuth Bayreuth Germany; ^12^ Centre For Muscle Research Department of Anatomy and Physiology The University of Melbourne Victoria Australia

**Keywords:** Helmholtz coil stimulation, magnetic microactuation, magnetic microspheres, mechanotransduction, myogenic differentiation, YAP signaling

## Abstract

Engineering skeletal muscle tissues with controllable bioactuation is essential for advances in biohybrid robotics, regenerative medicine, and high‐fidelity disease models. Mechanical stimulation has been shown to replicate the effects of physical exercise, while magnetic stimulation allows the manipulation of cells in a non‐invasive manner. Here, a platform based on Helmholtz coil pair for magnetic stimulation is developed. To focus the stimulation through mechanotransduction, magnetic microspheres (MMS) were conjugated to myoblast integrins at defined MMS‐to‐cell ratios, functioning as microscale actuators under alternating magnetic fields. Exposure of non‐labeled C2C12 cells to ∼2.9 mT, 50 Hz magnetic fields enhanced myogenic differentiation, with significantly increased fusion indices after 10 and 30 min of daily stimulation. Remarkably, MMS‐labeled cells (1:1 ratio) required only 2 min of daily stimulation to achieve comparable enhancement, demonstrating the efficacy of targeted microactuation. Mechanistic analysis revealed elevated nuclear localization of Yes‐associated protein (YAP) in stimulated MMS‐labeled cells, confirming activation of force‐dependent signaling pathways. qRT‐PCR analysis further supported these findings, showing stimulation‐associated upregulation of myogenic genes, particularly in MMS‐labeled cells. The integration of cell labeling with dynamic magnetic fields offers new opportunities for remote stimulation strategies in biofabrication, muscle tissue engineering, and therapeutic approaches for muscle tissue.

## Graphical abstract

A magnetic stimulation platform for the remote stimulation of cells is presented, consisting of a magnetic stimulator and magnetic microspheres. Skeletal muscle cells are labeled with magnetic microspheres, which act as local magnetic actuators on the cells upon application of the magnetic field. The platform offers great potential for non‐invasive control over cells and bioengineered tissues.

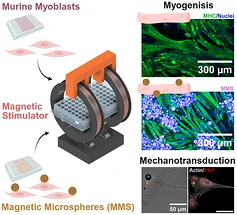

## 1 Introduction

Nature provides a broad spectrum of actuators, ranging from molecular motors to highly organized contractile tissues such as skeletal muscle [1, 2]. Inspired by these systems, engineered skeletal muscle has emerged as a key component in biohybrid robotics and as a promising platform for regenerative medicine, disease modeling, and cultured meat production [3, 4, 5, 6, 7, 8, 9]. A shared requirement across these applications is the availability of in vitro muscle models that are not only structurally developed but also functionally relevant. This challenge is particularly important in light of the increasing burden of musculoskeletal disorders in ageing populations and the continued lack of effective disease‐modifying therapies [10, 11, 12]. Yet conventional 2D culture systems do not adequately reproduce essential muscle functions, including contraction, fatigue, and adaptive responses to external stimuli [13]. Collectively, these limitations underscore the need for advanced in vitro platforms capable of supporting myogenic maturation and delivering controlled, non‐invasive, localized mechanical stimulation.

One of the most critical physiological cues driving muscle adaptation is exercise. Exercise profoundly affects the morphology and metabolism of multiple tissues, including muscle, bone, adipose tissue, and the nervous system [14]. While inactivity leads to atrophy and weakness, muscles retain a high capacity for recovery and adaptation [15]. Exercise enhances myoblast differentiation and myofiber remodeling through molecular pathways involving key transcription factors such as myogenin, myocyte enhancer factor 2 (MEF2), and myogenic differentiation factor (myoD) [16].

To replicate these exercise‐induced cues in vitro, biofabrication strategies increasingly incorporate external stimuli that mimic mechanical loading in the body [17]. Mechanical stimulation, for example, has been shown to promote myofiber development, as Powell et al. demonstrated through dynamic stretching of human myoblasts, which increased myofiber diameters and total myofiber area [17]. Another method capable of inducing mechanical loading is magnetic stimulation, an emerging approach that is an especially promising method due to its remote, non‐invasive nature. Magnetic actuation provides precise spatial and temporal control of cell behavior and can achieve mechanical effects without direct contact, offering advantages over traditional mechanical stimulation [15, 18].

Magnetic fields interact with cytoskeleton elements, membrane receptors, and ion channels, with cellular responses depending on field type and intensity [19, 20]. Such fields can be applied as static or dynamic, the former being mostly defined by the magnitude, while the latter is also defined by frequency [20]. Extremely low‐frequency electromagnetic fields (3 Hz to 3 × 10^3^ Hz) cannot break molecular bonds or generate heat but can induce weak electric currents in biological tissues [21]. Such time‐varying fields are beneficial for muscle stimulation because they better mimic the dynamic behavior of skeletal muscle [22, 23]. For example, Ayala et al. showed that only dynamic magnetic fields (50–70 mT), not static fields, increased cytosolic Ca^2+^ in myotubes from C2C12 murine myoblasts, after 2 min of exposure [24]. Additionally, some studies propose that magnetic fields may exert weak repulsive forces on cells due to the diamagnetic properties of cellular components [25].

Uniform magnetic fields can be generated using systems such as a Helmholtz coil pair or a Fanselau coil arrangement, where field strength and size are tuned by adjusting the superposition of fields from each coil [25, 26, 27, 28, 29]. Helmholtz coils are widely used for microrobot actuation [30, 31, 32] and have also been applied *in vivo* to modulate inflammatory responses [33, 34], as well as in vitro cell stimulations. Terrie et al. showed enhanced myogenesis in human skeletal muscle cells after daily exposure to a 2 mT and 75 Hz field [22]. Similarly, Yap et al. demonstrated that brief pulsed electromagnetic fields (PEMFs) stimulation (10 min, 0.5–3 mT, 15 Hz) promoted C2C12 myogenesis via transient receptor potential (TRP)‐C1–mediated calcium entry [23].

Magnetic fields can induce mechanical stimulation or heat through magnetic actuation [19, 35]. Mechanical cues may be applied indirectly by embedding magnetic microfillers into a polymeric matrix, where field‐induced deformation transfers forces to cells [35]. Several studies have used magnetically active hydrogels for this purpose [36, 37, 38, 39, 40]. For instance, Rios et al. incorporated magnetic particles into fibrin hydrogel and controlled the matrix motion with a linear actuator, improving C2C12 myotubes alignment and enhancing contraction [41]. Basak and Packirisamy similarly stimulated C2C12 cells in a porous ferrogel and observed increased myogenic marker expression, suggesting possible activation of Yes‐associated protein (YAP)‐mediated mechanotransduction, though no mechanistic data were provided [42].

Direct magnetic actuation applies forces to cells by physically or chemically attaching magnetic micro‐ or nanoparticles to individual cells, clusters, or organoids. External magnetic fields generate torques and attractive or repulsive forces between particles, which are transmitted directly to the attached cells [35]. This approach has been widely explored using internalized magnetic nanoparticles (MNPs) to control cell patterning, enhance tissue fabrication, or monitor engineered tissues [43, 44, 45, 46, 47, 48]. However, intracellular MNPs can pose cytotoxic risks, especially under alternating magnetic fields [49]. Their ability to selectively kill cancer cells underscores these potentially harmful effects [50]. Various strategies have been tested to reduce cytotoxicity, such as antioxidant coatings to protect erythrocytes [51]. Mechanisms of MNP‐induced cytotoxicity, including oxidative stress, genotoxicity, and membrane or cytoskeletal disruption, are reviewed elsewhere [52].

An elegant solution to avoid cytotoxicity associated with internalized magnetic particles is to attach them to the cell membrane rather than allow endocytosis. This can be achieved by using larger particles, which are less readily internalized [35]. For instance, Jafari et al. used 4.5 µm carboxylated magnetic microparticles to assemble fibroblasts and preadipocytes into 3D structures under static magnetic fields [53]. Several groups have also developed “magnetic backpacks” (4.75–7.75 µm) that attach to cell surfaces for phenotype modulation, magnetically guided positioning, drug release, and *in vivo* imaging [54, 55].

Membrane‐bound magnetic particles can transmit torque or forces to specific receptors when exposed to magnetic fields, thereby activating mechanotransduction pathways [35, 56]. Mechanotransduction converts mechanical cues into biochemical signals, influencing short‐term behaviors (adhesion, migration, and intracellular tension) and long‐term outcomes such as proliferation and structural organization [57, 58]. Chen et al. demonstrated this by attaching ligand‐coated ferromagnetic beads (diameter: ∼4.5 µm) to endothelial integrins and applying a twisting field, which upregulated endothelin‐1 expression [59]. Similarly, Maier et al. used Arg‐Gly‐Asp (RGD) functionalized microspheres (diameter: 1–10 µm) attached to C2C12 integrins and showed enhanced myogenesis following static magnetic stimulation (∼340 mT), with increased myogenic marker expression and fusion indices [60].

**FIGURE 1:**
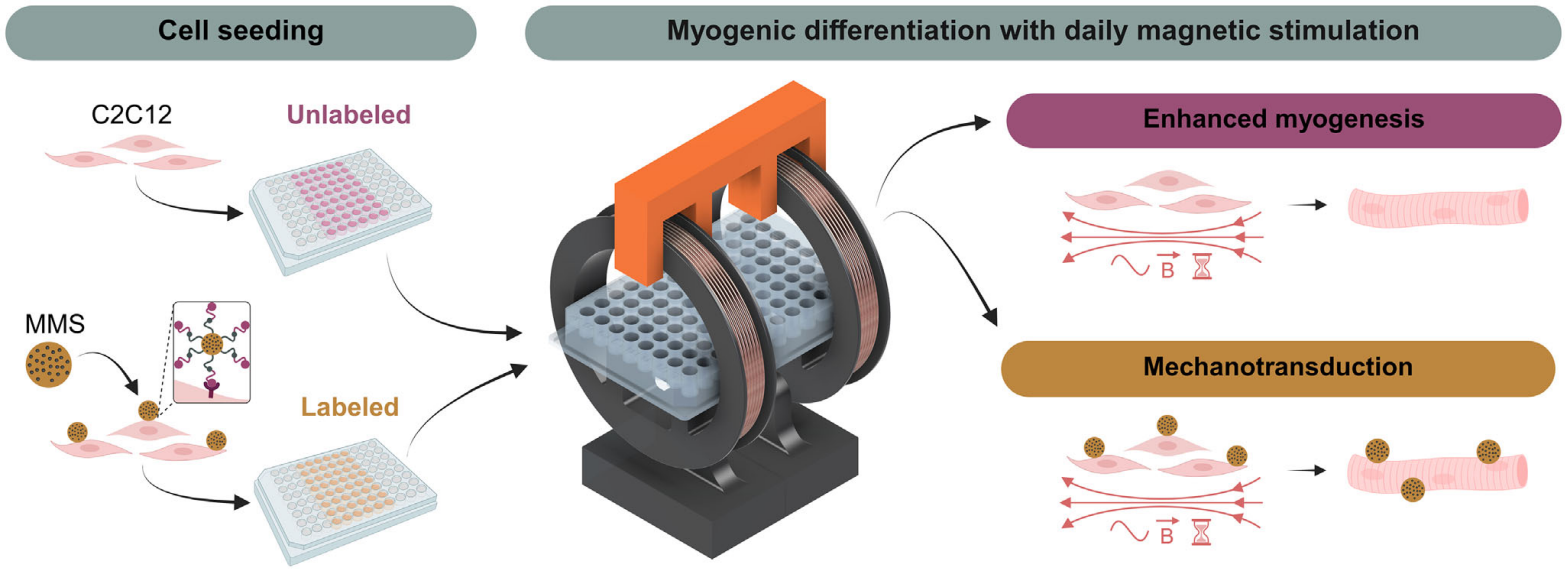
Workflow of this study. C2C12 muscle cells were stimulated with a dynamic magnetic field produced by a custom‐built magnetic stimulator to enhance myogenic differentiation. C2C12 muscle cells were further labeled with magnetic microspheres (MMS) on their integrin receptors to target the magnetic stimulation on individual cells through mechanical activation of mechanotransduction pathways. Created in BioRender. https://BioRender.com/jp5aiqo.

Here, we introduce a dynamic magnetic stimulation platform that combines a custom Helmholtz coil system with integrin‐targeted magnetic microspheres (MMS) to enable remote and localized mechanostimulation of skeletal muscle cells. The system generates uniform magnetic fields with tunable flux density, waveform, and frequency, providing programmable and reproducible stimulation across multiple culture formats. In contrast to previous designs in which the magnetic field is oriented perpendicular to the cell membrane [42], our platform applies the field parallel to the membrane, a geometry associated with stronger membrane current induction under low‐frequency conditions [61]. By coupling this field configuration with RGD‐functionalized magnetic microspheres bound to integrins, the platform concentrates magnetic forces at mechanosensitive sites rather than relying on bulk matrix deformation or intracellular nanoparticle uptake. Using this strategy, we investigated both myogenic differentiation and early mechanotransduction responses in unlabeled and microsphere‐labeled murine myoblasts exposed to extremely low‐frequency alternating magnetic fields (Figure 1). To our knowledge, this is the first study to combine dynamic Helmholtz coil‐based stimulation with integrin‐targeted magnetic microactuation in skeletal muscle cells, establishing a versatile framework to probe and control myogenesis through synergistic magnetic and mechanical cues.

## 2 Experimental Section

### 2.1 Materials

The electromagnetic stimulator was fabricated using copper wire (Stefan Maier GmbH, Germany) with a diameter of 0.6 mm, a lighting transformer (240 Vac to 12 Vac, 105 W) (ABN, Germany), a 3 Amp blade fuse (Eska, Germany), a screw terminal 30 A blade fuse holder, banana plugs, and banana sockets (Reichelt Elektronik, Germany). Chitosan 90/50 (HMC+, Germany), acetic acid (Fisher Scientific, UK), cottonseed oil (Acros Organics, Belgium), sorbitane trioleate (Span 85), iron oxide nanoparticle solution (1 mg/mL Fe, 30 nm, PEG‐functionalized, in H_2_O, Sigma Aldrich, US), and sodium tripolyphosphate (STPP, Sigma Aldrich, US) were purchased for the production of magnetic chitosan microparticles. Ethanol and acetone (VWR International, US) were used to separate the particles from the oil phase. Glycerol anhydrous (neoFroxx, Germany) was used for the drag test. Poly(ethylene glycol) (N‐hydroxysuccinimide 5‐pentanoate) ether N’‐(3‐maleimidopropionyl) aminoethane (NHS‐PEG‐MAL, Sigma Aldrich, US), 2‐(4‐(2‐Hydroxyethyl)‐1‐piperazinyl)‐ethane sulfonic acid (HEPES, Roth, Germany), sodium chloride (NaCl, VWR International, US), H‐Gly‐Arg‐Gly‐Asp‐Ser‐Pro‐Cys‐OH trifluoroacetate salt (GRGDSPC peptide, Bachem, Switzerland), trifluoroacetic acid (TFA, Iris Biotech, Germany), and ethylenediaminetetraacetic acid (EDTA, Roth, Germany) were purchased for functionalization of the particles.

For biological studies, C2C12 murine myoblasts (ACC 565, DSMZ, Germany) were used. Reagents used in experiments are listed as follows: Dulbecco's modified eagle medium (DMEM) – high glucose with L‐glutamine, Sigma‐Aldrich, US), fetal calf serum (FCS) (Bio & Sell, Germany), Pen/Strep (Thermo Fisher Scientific, US), horse serum (Thermo Fisher Scientific, US), Insulin‐Transferrin‐Selenium (ITS, Thermo Fisher Scientific, US), PROFUSE‐S1 (ProFuse Technology, Israel), Alamar Blue stock solution (3 mM, resazurin sodium salt, Merck, Germany), 50% glutaraldehyde (Roth, Germany), paraformaldehyde (PFA, Roth, Germany), and Triton X‐100 (Roth, Germany), Phalloidin‐Tetramethylrhodamine B isothiocyanate (Phalloidin‐TRITC, Merck, Germany), and 4',6‐diamidino‐2‐phenylindole (DAPI, Merck, Germany), Dulbecco's phosphate buffered saline (PBS, Bio & Sell, Germany), bovine serum albumin (BSA, Roth, Germany), primary antibodies YAP1 (Mouse monoclonal IgG_2_ Antibody (63.7), #101199, Santa Cruz Biotechnology, US) and MF20 (Myosin 4 Monoclonal Antibody, #14‐6503‐82, Invitrogen, US), and secondary antibody Alexa Fluor 488 goat anti‐mouse IgG (H+L) (Thermo Fisher Scientific, US). Kits used for RNA extraction and qRT‐PCR are: innuPREP RNA Mini Kit 2.0 RNA (845‐KS‐2040050, Analytik Jena, Germany), Revert Aid RT Kit (K1691, ThermoFisher Scientific, Germany), and Innumix qPCR DSGreen mix (845‐AS‐1320500, Innuscreen GmbH, Germany).

### 2.2 Magnetic Stimulator Construction and Evaluation

The magnetic stimulator was designed using Fusion 360 software (version V.2.0.15775). Circular frames were 3D printed (Pro3, Raise3D, US) using ABSpro Flame Retardant (black, 1.75 mm, FormFutura, Netherlands) with the following printing parameters: printing temperature 250°C, print bed temperature 110°C, extrusion speed 30 mm/s, and layer thickness 0.1 mm. Post‐printing, support materials were meticulously removed using tweezers and a box cutter. To form the electrical coils, the transformer wire was first coiled around one frame 200 times. The wire was then extended to the second frame and similarly wrapped to match the number of turns of the first coil. Ensuring that the wire winds in the same direction on both coils is crucial, as this allows the current of both coils to flow in the same direction. This aligned flow leads to the superposition of both magnetic fields, ultimately causing a uniform magnetic field at the center between the coils. The wire ends were stripped carefully with a soldering iron and tin solder at 400°C, removing the insulation and forming an electrical contact with the power source. The ends of the wire were fastened into banana sockets, which in turn were adhered to the 3D‐printed frameworks. A fuse was installed between the commercially available lighting transformer, used as a power source, and the device to limit the current to a maximum of 3 A, thereby ensuring safe operation. Banana plugs were soldered to the cable ends that would be attached to the Helmholtz coil pair. A printed clamp keeps a defined distance between the coils and thereby ensures a stable magnetic field. Well plates containing cells can be positioned in the center of the circular frames so that the cells are exposed to the magnetic field. Images of the fabrication process, step‐by‐step, can be found in the Supporting Information (Figure S1).

To determine the magnetic flux density in the magnetic stimulator, three different methods were applied and compared as follows: First, the magnetic flux density B in the center between the coils was calculated with Equation 1 [31, 62, 63].

(1) $$B=N\cdot I\cdot \mu \cdot \left[\frac{r^{2}}{{\left(r^{2}+\frac{L^{2}}{4}\right)}^{\frac{3}{2}}}\right]$$

where *N* is the number of wire turns, *I* in A is the current running through the coils, *r* in m is the radius of the coils, *L* in m is the distance between the coils, and the permeability *µ* is *µ = µ_r_ × µ_0_*. The relative permeability *µ_r_* was assumed to be 1, and the permeability of free space *µ_0_* is 4π × 10^−7^ N A^−2^ [64]. Second, to simulate the magnetic field in the horizontal plane between the Helmholtz coils, a MATLAB code for Finite Element Method Magnetics (FEMM) was developed (in MATLAB version R2025b, FEMM 4.2) that builds and solves the problem. The code can be found in the Supporting Information (**Code S1**). Third, the magnetic flux density between the coils was measured with a Teslameter (Koshava 3, Wuntronic, Germany) at different locations in the field. The current (AC) was measured by connecting a digital multimeter (HT118C, Tilswall, China) in series with the system. The measurement was done every 15 min for 1 h. To mitigate any safety concerns that could arise from heating the coil's wire due to the flow of electric current, the wire temperature and medium temperature in the well plate were closely monitored for 1 h using a thermal imaging camera (B335 Thermal Imager, Teledyne FLIR, Germany). The temperature of transformer wire was recorded every 30 s during the first 10 min and every 1 min thereafter. The well plate temperature was measured at 10 min intervals, starting from the first 10 min.

### 2.3 Production of Magnetic Microspheres

The chitosan MMS are produced using a modified emulsion crosslinking technique reported by Anal et al. [65]. Briefly, a 2% (w/v) chitosan solution in 1% (v/v) acetic acid was filtered by passing through 0.45 µm and 0.2 µm filters to remove any debris and impurities. An oil bath was prepared by mixing 1.2 mL of Span 85 (0.8% (v/v)) into 150 mL of cottonseed oil in a 250 mL glass beaker at 37°C. A volume of 7.5 mL of the chitosan solution was vortex‐mixed with 1.69 mL of the iron oxide nanoparticle suspension for 20 s. Acetone (10 mL) was gradually added to the solution, which was vortexed again, and 9 mL of it was then slowly added to the oil bath while stirring with a magnetic stirrer at 800 rpm. Stirring was continued at 37°C and 800 rpm overnight. Subsequently, 1 mL of 28.8 mg mL^−1^ STPP was added to the emulsion and stirred continuously for 5 h, first at 800 rpm for 3 h and then at 600 rpm for 2 h. The oil‐microsphere suspension was centrifuged (524 g, 10 min, 5°C) (Multifuge X Pro Series, Thermo Fisher Scientific, US) to separate the MMS from the oil mixture. The MMS sediment was sequentially washed three times each with pure acetone, 100% ethanol, 40% ethanol, and lastly deionized water (diH_2_O) before being stored in diH_2_O at 4°C.

#### 2.3.1 Characterization of the Magnetic Microspheres

##### 2.3.1.1 Morphology, Size, and Shape

The morphology of the MMS, their size, and shape were evaluated using bright‐field microscopy (DMI 3000B, Leica, Germany), confocal fluorescence microscopy (TCS SP8 HyD, Leica, Germany), and scanning electron microscopy (SEM) (Volumescope, Thermo Fisher Scientific, US) at different magnifications. For SEM, the MMS were imaged after air‐drying the homogeneous particles on a glass slide, sputter‐coated with platinum. The diameters of more than 350 MMS were measured in fluorescence images using Fiji (version 2.9.0).

##### 2.3.1.2 Magnetic Properties

The color differences of the microspheres with and without MNPs were evaluated macroscopically using a mobile phone camera. To confirm the incorporation of iron oxide nanoparticles into the chitosan microspheres, complementary X‐ray diffraction (XRD) and small‐angle X‐ray scattering (SAXS) analyses were performed. XRD was carried out using a D8 Discover diffractometer (Bruker, Germany), equipped with a Cu‐K_α_ monochromatic X‐ray source and configured in Bragg‐Brentano geometry. Scans were conducted over a 2θ range of 10° to 90°, in coupled theta/two‐theta mode with a step size of 0.009° and a scan rate of 10 steps per minute. The sample powder was mounted onto a zero‐background holder and secured using a minimal amount of pharmaceutical‐grade vaseline to ensure optimal adherence and prevent particle displacement during analysis. Moving average smoothing with 20 data points was applied. SAXS analysis was performed on both the MMS and MNP suspensions using a Double Ganesha Air system (SAXSLAB/Xenocs, France), featuring a PILATUS 300K position‐sensitive detector (Dectris, Switzerland) and a rotating anode X‐ray source (*λ* = 1.54 Å, Cu‐K_α_, MicroMax 007HF, Rigaku Corporation, Japan), under ambient conditions. The samples were measured either in air or within 1 mm glass capillaries (Hilgenberg, Germany) in water, with variable sample‐to‐detector distances to cover an extended range of scattering vectors *q*. Intensity profiles *I*(*q*) versus *q* were obtained through radial averaging with the instrument's proprietary software. Background scattering from air or Milli‐Q water‐filled capillaries was subtracted as appropriate. Theoretical intensities were calculated using Scatter software (version 2.5) [66].

Furthermore, the movement of magnetic microspheres exposed to a permanent magnet was monitored. For this, the microspheres' magnetic volume susceptibility was determined through a drag test measuring the particles' velocity toward a permanent magnet. The MMS were dried and redispersed in a 35% (v/v) glycerol/water solution. A 5 mm channel created between two stacks of two‐layered double tape on a microscope slide was used to monitor the movement of 30 µL of the microsphere suspension into the channel. The slide was placed on a microscope with a 10x/0.22 magnification objective placed 5 mm away from the slide edge. Then, an N45 neodymium magnet (20 mm diameter and 4 mm wide) was placed against the channel edge. The schematic of the setup is shown in the Supporting Information (Figure S2A), and a representative video of the MMS movement is shown in Supporting Information Video S1. The velocity of microsphere movement was calculated by dividing the distance traveled by the time using Fiji software (version 2.9.0). The magnetic Kelvin force, *F_K_*, acting on the spheres, was determined using Stokes’ Law for the drag force, *F*_S_, shown in Equation 2 [67].

(2) $$F_{K}=-F_{S}=6\cdot \pi \cdot r\cdot \eta \cdot v$$

where *F_K_* in N is the magnetic force acting on the specific MMS, *r* in m is the radius of that MMS, *η* in Ns m^−2^ is the dynamic viscosity of the solution, and *v* in m s^­1^ is the velocity of the MMS. The dynamic viscosity of the glycerol/water mixture (3.1984 10^−3^ Ns m^−2^) was determined using an online calculator by Chris Westbrook from the University of Reading [68]. The magnetic susceptibility of each magnetic chitosan microsphere in the drag test is calculated using Equation 3 **[** 69].

(3) $$\chi_{i}=\frac{\vec{F_{K}}\cdot \mu_{0}}{V\cdot \vec{B}\cdot \left(\nabla \vec{B}\right)}$$

where *V* in m^3^ is the volume of the MMS (calculated from diameter measurements taken via microscopy (DMI 3000B, Leica, Germany)), *µ_0_* is the permeability of free space (4π × 10^−7^ N A^−2^), $\vec{B}$ in T is the known applied magnetic field, and $\nabla \vec{B}$ in T m^−1^ is the gradient of that field [69]. Both $\vec{B}$ and $\nabla \vec{B}$ were determined using finite element modeling data from FEMM software and confirmed via field measurements utilizing a Teslameter, with values of 116.82 mT at the position (*x*, *y*) = (0, 6.5) in mm from the magnet's center and 9.4 T m^−1^ calculated in the y direction between the positions (0, 2) and (0, 7.5). A plot illustrating the magnetic field distribution is provided in the Supporting Information (Figure S2B).

### 2.4 In Vitro Cell Culture Studies

Murine C2C12 cells were maintained in a growth medium consisting of high‐glucose DMEM, 10% (v/v) FCS, 1% (v/v) Pen/Strep, and 20 mM HEPES at 37°C in an atmosphere of 5% CO_2_. Differentiation of the labeled cells was induced when they reached 95–100% confluency by adding a differentiation medium composed of high‐glucose DMEM with 2% (v/v) horse serum, 1% (v/v) Pen/Strep, 20 mM HEPES, and 1% (v/v) ITS, which was freshly added to the medium each time. The differentiation medium was exchanged every day after washing the cells once with PBS. Furthermore, the PROFUSE‐S1 supplement was added only for the first 24 h of differentiation in all samples at a dilution of 1:1000 in differentiation medium. Comparative data of the effect of PROFUSE‐S1 on C2C12 differentiation for 3, 4, and 5 days are shown in the Supporting Information (Figure S3).

#### 2.4.1 Labeling of C2C12 Cells with Magnetic Microspheres

The MMS were functionalized with RGD peptide, according to Maier's protocol [60]. Briefly, 1 mL of MMS suspension was evenly functionalized after homogeneous dispersion using vortexing. To prepare a linker to the MMS, 6.4 mg of NHS‐PEG‐MAL was dissolved in 1 mL of buffer (10 mM HEPES, 150 mM NaCl, pH 7.2), creating a final solution with a 2 mM concentration, which was mixed with centrifuged MMS (15 000 g, 10 min, 5°C) (Fresco 17, Thermo Scientific, US), collected as a pellet. The linker suspension was gently rotated on an Intelli Mixer (20 rpm) for 1 h to allow the linker to bind to the MMS. Following another centrifugation and removal of the supernatant, a peptide solution was introduced. This solution was composed of 45.4 µL of a 1.1 mg/mL GRGDSPC peptide stock solution in 0.1% (v/v) TFA, mixed into 1 mL of buffer (10 mM HEPES, 150 mM NaCl, 5 mM EDTA, pH 6.8). The suspension was then left to react on the Intelli Mixer (20 rpm) for at least 5 h. After the functionalization process, the MMS were sterilized by three washes with 80% ethanol, followed by three washes with growth medium to remove any residual ethanol. The MMS were stored in a growth medium at 4°C for future use. For cell labeling, the MMS suspension was vortexed to disperse the MMS and added to C2C12 myoblast cells at a ratio of 6:1 (6 MMS per cell) or 1:1 (1 MMS per cell). The mixture of cells and MMS was then topped up to the desired total volume with a fresh growth medium and incubated at 37°C for 30 min with a gentle rotation after 15 min.

#### 2.4.2 Evaluation of Labeled C2C12 Cells

First, the metabolic activity of cells was measured at different time points (1, 2, 3, and 4 days of culture) and different ratios of MMS to cells (6:1 and 1:1) compared to an unlabeled control. To quantify the proliferation of labeled cells compared to unlabeled cells, an Alamar Blue assay was performed at each time point. For this assay, 1000 cells/well were cultured in a cell culture‐treated 96‐well plate and incubated in a growth medium for 24 h, which was then replaced by 100 µL of 1:60 diluted Alamar Blue stock solution at each time point and incubated for 2.5 h. Following incubation, cell metabolic activity was measured using a plate reader (Mithras LB 940, Berthold Technologies, Germany) at an excitation wavelength of 560 nm and an emission wavelength of 590 nm. At least nine replicates were used for each condition and time point. The same protocol was used to evaluate the metabolic activity of unlabeled and labeled (1:1) cells under no stimulation and at 2, 10, and 30 min daily magnetic stimulation.

Second, to evaluate the morphology of the labeled C2C12 cells (both 6:1 and 1:1 ratio) with SEM, labeled cells were cultured on circular glass slides overnight and then fixed for 1 h using 2.5% (v/v) glutaraldehyde, and progressively dehydrated by sequentially applying a graduated series of ethanol concentrations (50, 70, 80, 90, and 100% (v/v)) for 10 min each. After lyophilization, the samples were sputter‐coated and imaged with SEM.

Third, the potential uptake of MMS by cells was evaluated. For this purpose, cells labeled with a 1:1 ratio (1 MMS per cell) were seeded on ibidi‐treated µ‐Slide 8‐Well slides, fixed using 3.7% (v/v) PFA for 15 min at room temperature (RT), and permeabilized with 0.1% Triton X‐100 solution for 5 min at RT. The cells were stained for their actin filaments with 100 µL of 200 nM Phalloidin‐TRITC solution and left to react in the dark for 30 min. Then, 100 µL of 300 nM DAPI solution was added, and the samples were incubated for 5 min at RT. After washing with PBS, the samples were imaged by confocal microscopy using z‐stack analysis.

#### 2.4.3 Magnetic Actuation of MMS on Labeled C2C12

To analyze the capacity of the MMS to act as a mechanical stimulus inside a dynamic magnetic field, C2C12 cells were labeled with a 1:1 ratio of MMS to cells as described before and then seeded on ibidi‐treated µ‐Slide 8‐Well slides at a density of 10^4^ cells/well. After 24 h of culture, 2 min of dynamic magnetic stimulation was applied on two consecutive days. All wells were positioned within the central region of the magnetic stimulator, where the magnetic field was most uniform. After the last stimulation, the cells were fixed using 3.7% (v/v) PFA for 15 min at RT, and permeabilized with 0.1% Triton X‐100 solution for 5 min at RT. Subsequently, to prevent non‐specific binding, the samples were blocked with 5% (w/v) BSA for 30 min at 37°C. This was followed by overnight incubation in primary antibody YAP1 (targeting YAP, in a 1:100 dilution in 1% (w/v) BSA) in the dark at 4°C. Subsequently, the samples were washed three times with PBS, and the binding of the primary antibody was revealed through incubation of the samples with a secondary antibody (Alexa Fluor 488 goat anti‐mouse IgG (H+L), in a 1:500 dilution in 1% (w/v) BSA) for 1 h at RT in the dark, followed by Phalloidin‐TRITC and DAPI staining as described above. The samples were imaged by confocal microscopy. Samples without stimulation were used as a control. The images were analyzed using Fiji (version 2.9.0). The mean grey value of the YAP channel was segmented in nuclei and cytoplasm to calculate the nuclear/cytoplasmic YAP ratio. From both labeled and unlabeled cells (on which MMS didn't attach), 15 cells per group were analyzed.

#### 2.4.4 Magnetic Stimulation of Unlabeled and Labeled C2C12 Cells Throughout Differentiation

##### 2.4.4.1 Daily Magnetic Stimulation

To analyze the impact of daily magnetic stimulation on C2C12 cells, unlabeled and labeled cells (1:1) were seeded on ibidi‐treated µ‐Slide 8‐Well slides at a density of 10^5^ cells/well. After 24 h of culture (95–100% confluency), magnetic stimulation was applied once every day for different periods of 2, 10, and 30 min. The stimulation was applied at RT, after which the cells were immediately transferred back to 37°C. In a control group, the cells were not stimulated, but also placed at RT for the stimulation periods to ensure similar conditions for all plates. All wells were positioned within the central region of the magnetic stimulator, where the magnetic field was most uniform. The samples were exposed to stimulation for 4 days. Cells were kept in a differentiation medium after the first 24 h of culture.

##### 2.4.4.2 Immunocytochemistry and Analysis

On day 5 of culture, the samples were evaluated for expression of myosin heavy chain (MHC) and formation of multinuclear myotubes. Therefore, the samples were first fixed with 3.7% (v/v) PFA and permeabilized with 0.3% (v/v) Triton X. Subsequently, to prevent non‐specific binding, the samples were blocked with 5% (w/v) BSA for 30 min at 37°C. This was followed by overnight incubation in primary antibody MF20 (targeting Myh4, in a 1:500 dilution in 0.1% (w/v) BSA) in the dark at 4°C. Subsequently, the samples were washed two times with PBS and incubated again in a mixture of a secondary antibody (Alexa Fluor 488 goat anti‐mouse IgG (H+L) with a 1:500 dilution in 0.1% (w/v) BSA and DAPI (18 nM) for 1 h at RT in the dark.

For each experimental group, four technical replicates were examined using a confocal microscope, capturing four different fields of view for each replicate. These images were analyzed using Fiji (version 2.9.0). Fusion indices were computed by quantifying the nuclei in myotubes with more than two nuclei and comparing them to the number of nuclei not associated with myotubes. The collective count in each experimental group was used to calculate the fusion index using Equation 4 [70].

(4) $$Fusion\;index\;\left[\%\right]=\frac{nuclei\;of\;multinucleated\;myotubes\;\left(\geq 2\right)}{all\;nuclei}\cdot 100$$

Myotube diameter was measured at two points along each myotube in one image per replicate. For each experimental group, the mean diameter was calculated by first averaging the measurements within each replicate and then averaging the replicate means.

##### 2.4.4.3 RNA Extraction and qRT‐PCR

Furthermore, the myogenesis gene expression in cells (unlabeled and labeled) after the daily stimulations was analyzed after collection via trypsinization, pelleting in microcentrifuge tubes, washing with DPBS, re‐pelleting, and storing at −80°C. For the RNA extraction, an innuPREP RNA Mini Kit was used according to the manufacturer's instructions on the pelleted cells. RNA concentration was determined with a Nanodrop, and 2.5 µg were used to synthesize cDNA with the Revert Aid RT Kit according to the manufacturer's instructions. qRT–PCRs were performed on 1 µl of cDNA with an Innumix qPCR DSGreen mix on a qTower 2.0 qRT–PCR cycler (Analytik Jena). Each set of primers was verified through a two‐step verification protocol. The first step included the testing of nine combinations of primer concentrations against 50 ng of cDNA from differentiated C2C12 cells. The combination that gave the lowest C_T_ value, a single peak in the melt curve, and only one product at the expected size when the qPCR reaction was run on a 2% agarose gel was chosen for the second step of the verification. During this, the linearity of the C_T_ values given by the primer combination identified in the first step against a serial cDNA dilution ranging from 250 to 7.8 ng was compared to the linearity of the C_T_ values of the housekeeping gene (for these experiments, Ywhaz [71]). The primers used and their concentration are shown in Supporting Information Table S1.

### 2.5 Statistical Analysis

Given the limited sample size, normality was not assumed, and formal normality testing was not used to determine the statistical approach. Therefore, unless otherwise stated, data are reported as median (interquartile range (IQR)) from at least three independent replicates. Statistical analyses were performed using non‐parametric methods, as appropriate for each comparison. Differences among more than two groups were assessed using the Kruskal–Wallis test with Dunn's post hoc multiple‐comparison test, while differences between two groups were assessed using the Mann–Whitney test. Levels of statistical significance are indicated as follows: p < 0.05 (*), p < 0.01 (**), and p < 0.001 (***). Analyses were performed using GraphPad Prism version 11.

## 3 Results and Discussion

### 3.1 Design and Evaluation of the Magnetic Stimulator Device

**FIGURE 2:**
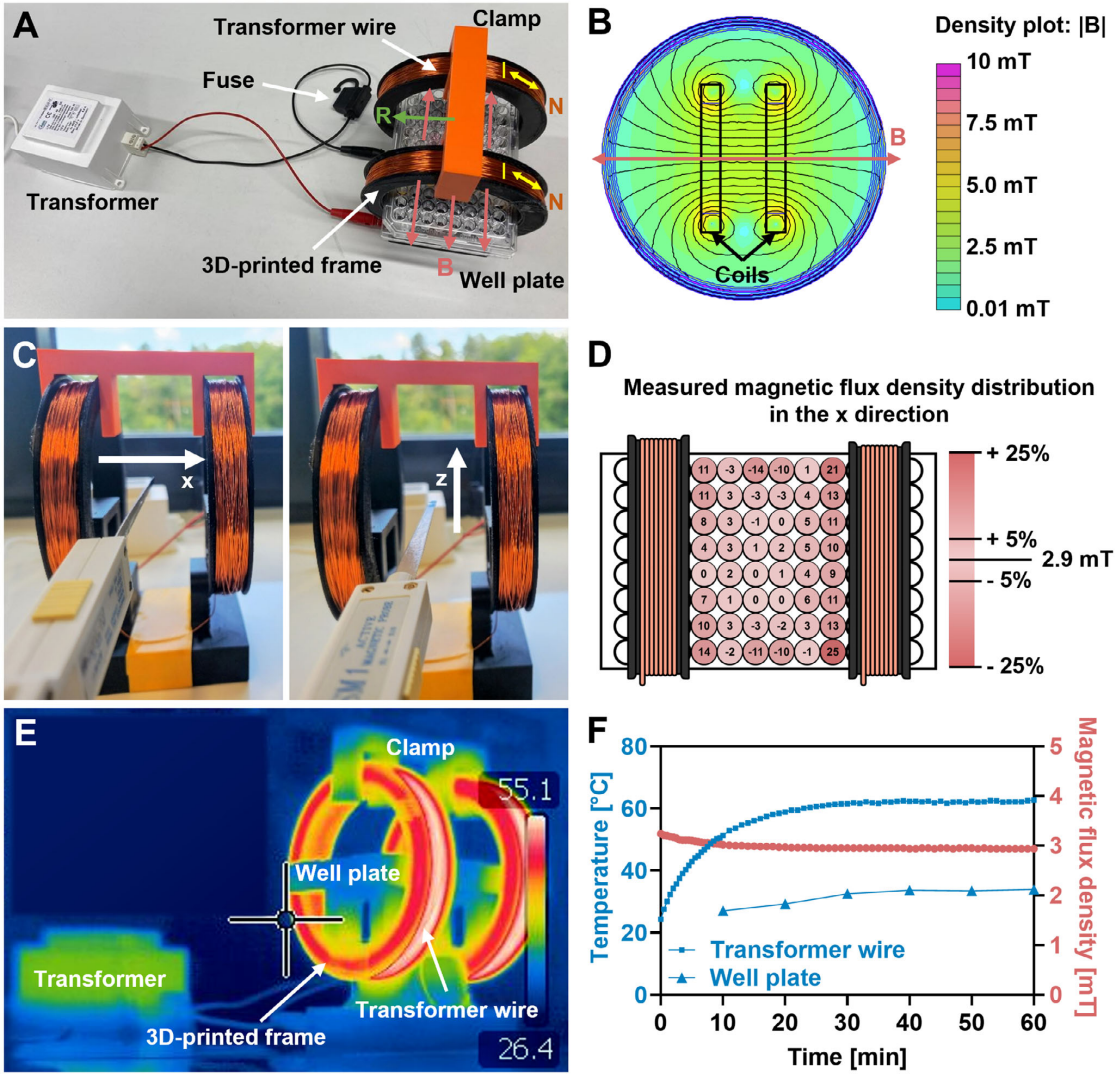
Electromagnetic stimulator. (A) Picture of the electromagnetic stimulator showing the transformer, 3D‐printed frame with transformer wire, fuse, clamp, and a well plate. (B) Finite Element Method Magnetics (FEMM) simulation of the magnetic flux density direction and distribution. (C) Measurement of the magnetic flux density in the *x* and *z* directions using a Teslameter. (D) Measurement of the magnetic flux density distribution in the *x* direction of the applied magnetic field in a 96‐well plate, measured in the 48 wells between the coils. (E) The temperature of all parts of the magnetic stimulator after 10 min was captured by a thermal imaging camera. (F) Magnetic flux density in the center between the coils and maximum temperature over time (measured on the surface of the transformer wire and on the well plate).

A versatile electromagnetic stimulator employing the principle of the Helmholtz pair of coils was designed (Figure 2A). Helmholtz coils consist of two similar, parallel coaxial coils, each with radius *R* and number of turns *N*, which are placed at a distance equal to the radius of one coil. If a current *I* runs through the coils, a uniform magnetic field *B* is produced in the space between them. Along the *z*‐axis of the coils, the magnetic field can be calculated using the superposition principle by adding up the individual magnetic fields of the coils [28, 30, 33]. To provide an alternating current to the system, a commercially available lighting transformer was used as a cost‐effective power source. It transforms the voltage provided by sockets (240 V, 50 Hz) down to 12 V. The frequency remains at 50 Hz, which is transferred directly from the transformer to the magnetic stimulator. Other power sources, like function generators, can be used to tune the frequency, waveform, and amplitude of the current and the resulting magnetic field.

The prototype was designed with *N* = 200 turns, *r* = 5 cm, and *L* = 7 cm, as shown in the Supporting Information (Figure S4). The distance between the coils was increased; by expanding the coil spacing, the length of the uniform field area may be extended, although with a minor reduction in the maximum field strength [29, 63]. The current from the lighting transformer was measured to be 1.22 A at the start of the measurement. Using these parameters, the magnetic flux density in the center between the coils was calculated with Equation 1 to be 3.37 mT. Further, the magnetic field was simulated in FEMM to obtain the direction and distribution of magnetic flux density B, yielding an expected magnetic flux density of 3.0–3.5 mT in the central area (Figure 2B). Lastly, a Teslameter was used to measure the magnetic flux density, which was 2.9 mT in the center in the *x* direction, while it was 0.09 mT in the *z* direction, confirming the field directionality (Figure 2C). It was observed that, in the *x* direction, the magnetic flux density does not deviate by more than 5% within the central 24 wells of a 96‐well plate (Figure 2D), showing that the magnetic flux density is very uniform in this area. Accordingly, all cell culture experiments were performed within this central area. This uniformity of magnetic flux density in the center of the Helmholtz coils is in agreement with the literature, as previously shown by Haghnegahdar et al. [33].

The heat development of the Helmholtz coils and the medium in the well plate was monitored with a thermal imaging camera over time to ensure safety for the user as well as the stimulated living cells. Figure 2E shows an exemplary thermal image of the coils and the transformer after being turned on for 10 min. More images taken of the magnetic stimulator at different time points are shown in the Supporting Information (Figure S5). In Figure 2F, the temperature of the transformer wire and the well plate are plotted over time. During the initial 30 min, the temperature of the transformer wire rises continuously, reaching a plateau at 62°C. To ensure that the 3D‐printed circular frame can endure this temperature, acrylonitrile butadiene styrene (ABS) was chosen as the printing material due to its glass transition temperature of 105°C, which is more than 40°C higher than the maximum temperature of the coils, and due to its stable mechanical properties below this glass transition temperature [72]. The temperature of the medium in the well plate (Figure 2F) was also measured and never exceeded 34°C, ensuring that the cells cultured in the medium are at a safe temperature during stimulation.

The heat development of the transformer wire further affects the magnetic properties of the system. The magnetic flux density measured with a Teslameter over time is also plotted in Figure 2F. It shows a decline in the first 30 min, starting at 3.25 mT and settling at a final value of 2.94 mT. This decrease in magnetic flux density alongside a rise in wire temperature can be attributed to the temperature sensitivity of the copper wire in the coils, which is a known phenomenon [73]. Starting with an initial current of 1.22 A at RT (23°C), the current decreases to 1.12 A after 30 min and remains constant for the final 30 min. The calculation by Equation 1 using this current reveals a decreased magnetic flux density of 3.1 mT at 62°C (30 min) compared to the magnetic flux density of 3.37 mT calculated for RT, proving that the measured decrease in magnetic flux density can be explained theoretically. Therefore, the magnetic stimulator was always switched on for 30 min before starting the magnetic stimulation of cells to ensure a stable magnetic flux density.

Previously reported commercial dynamic Helmholtz coil stimulators (for example, HHS 5201–98, Schwarzbeck Mess‐Elektronik, Germany) have been used at magnetic flux densities of 1–1.5 and 3 mT with frequencies of 40 Hz and 5 Hz, which is in the same order of magnitude as our work [36, 37]. A downside of these systems is that they are very expensive (multiple thousand euros) compared to the custom‐built device presented in this work (less than 200 euros). Here, we provide step‐by‐step instructions on how to successfully design, simulate, and build such a system with appropriate dimensions to ensure safety. A further benefit of our system is its flexibility. The system is compatible with different power sources, enabling the generation of either static magnetic fields using a direct current (DC) power supply or dynamic magnetic fields using an alternating current (AC) power supply, as employed in the present study. Using a function generator instead of the transformer, the waveform and magnetic flux density of the dynamic field can easily be tuned.

### 3.2 Magnetic Microspheres Production and Properties

One of the main aim of this study was to investigate the impact of a dynamic magnetic field applied to magnetically labeled muscle cells. To do so, we first investigated the homogeneous production of chitosan microspheres containing MNPs. The MMS were produced with an oil‐emulsion method and crosslinked with the ionic crosslinker STPP, a non‐toxic polyanion interacting with the polycation chitosan by electrostatic forces [74].

**FIGURE 3:**
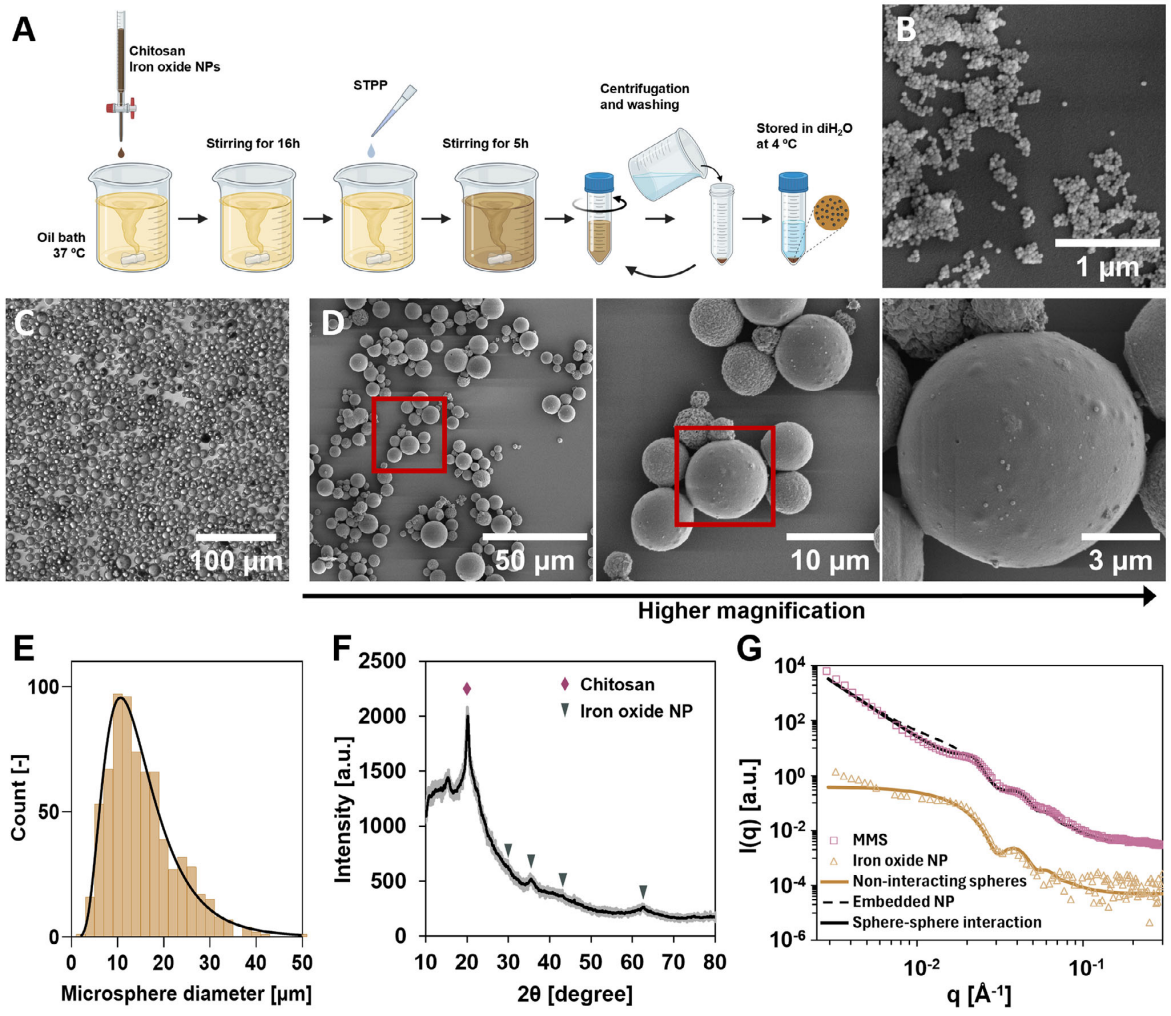
Production and characterization of magnetic microspheres (MMS). (A) Schematic of the fabrication of MMS. Created in BioRender. https://BioRender.com/v09qqsi (B) SEM image of magnetic nanoparticles (MNPs). (C) Bright‐field image of MMS. (D) SEM images showing the MMS at different magnifications. (E) Size distribution of MMS after analyzing fluorescence images. (F) XRD pattern showing the crystalline phases of MMS with a moving average smoothing with 20 data points. (G) SAXS of MMS and MNPs compared to theoretical models.

Figure 3A shows an overview of the MMS production process. The MNPs (Figure 3B) were encapsulated in the chitosan microspheres by adding them to the chitosan solution before particle formation. The resulting MMS were imaged using optical microscopy (Figure 3C) as well as SEM (Figure 3D) to determine their size and shape. Figure 3E shows that the size distribution of MMS fits a lognormal distribution; therefore, the mean microsphere diameter is 13.6 ± 1.6 µm (geometric mean ± geometric standard deviation). This diameter is within the targeted size range between 5 µm, to avoid endocytosis [53], and 30 µm C2C12 cells' size [75]. Further, the microscopy images demonstrated that most MMS are perfectly spherical (Figure 3C,D), confirming the success of the fabrication method.

In addition to the morphology of MMS, their magnetic properties and encapsulation of the MNPs in MMS were evaluated using multiple methods: 1) a color comparison, 2) XRD, 3) SAXS, and 4) attraction of the MMS to a permanent magnet. First, the particles were visually analyzed after synthesis. Shown in Supporting Information, Figure S6A, the color of microspheres without and with encapsulated MNPs was compared, showing that the microspheres without MNPs have a brown color, while the MMS have a black color due to the encapsulated MNPs.

We further structurally analyzed the MMS to detect the MNPs embedded in their structure. The XRD pattern of the magnetic microspheres (Figure 3F) displays characteristic peaks at 2θ = 20.1°, 30.1°, 35.5°, 43.3°, and 62.8°. The presence of a peak at 2θ = 20.1° indicates the presence of chitosan, whereas the peaks at 2θ = 30.1°, 35.5°, 43.3°, and 62.8° are associated with the MNPs. These peaks can be assigned to the diffraction of the (2 2 0), (3 1 1), (4 0 0), and (4 4 0) planes, respectively, of spinel‐structured magnetite nanoparticles (JCPDS No. 19–629) [76, 77, 78].

To substantiate the observations obtained via SEM and XRD, additional SAXS measurements were conducted on solid MMS samples, and for reference, on the iron‐oxide magnetic nanoparticles (MNPs) used in their preparation (Figure 3G). The scattering intensity *I(q)* of the MMS exhibits a *q^−4^* dependence at low scattering vectors (*q* < 0.01 Å^−1^), followed by oscillations. The *q^−^* ^4^ scaling reflects Porod's law, consistent with sharp, unresolved interfaces of micron‐sized spheres whose individual surface features lie well below the resolution of the SAXS experiment. The scattering pattern for the MNPs is flatter in the low *q* region and reveals a characteristic oscillation minimum around q ≈ 0.03 Å^−1^, indicative of the MNP's size. Modeling the scattering intensity of the MNPs using a non‐interacting sphere model (yellow line) yielded an effective MNP diameter of 29 ± 3 nm, confirming the 30 nm diameter specified by the manufacturer. Deviations from the theoretical intensity at low *q* hint at inter‐particle interactions or initial clustering, in line with the agglomerates visible by SEM (Figure 3B). In the first approximation, the MMS scattering profile is described by adding the *q^−4^* contribution and the scattering component of single‐dispersed non‐interacting MNPs (dashed black curve). A significantly improved description, especially in the range of q ≈ 0.02 Å^−1^, is obtained when inter‐particle interactions among the embedded MNPs are included through a hard‐sphere Percus–Yevick structure factor with an effective inter‐particle distance of 29 nm, and a volume fraction of 0.35. The total model (solid black line) then consists of the scattering contributions of homogeneous micron‐sized spheres (outer phase, which contributes mainly to the low *q*) and that of interacting small MNPs (inner phase). This observation suggests that clustered aggregates of MNPs may be transferred from the iron oxide nanoparticles suspension into the MMS during synthesis, resulting in the incorporation of individual MNPs (approximately 30 nm in diameter) alongside potential regions of agglomerates within the chitosan microspheres.

Furthermore, the attraction of MMS to a permanent magnet was observed (Supporting Information, Figure S6B). To quantify the magnetic attraction in the MMS, we conducted a drag test using a self‐made setup adapted from Grob et al. [67], yielding a magnetic susceptibility range of 0.002 to 0.054, with a mean value of 0.013 ± 0.012 and a median of 0.007. The relatively high standard deviation likely reflects heterogeneity in the distribution and concentration of MNPs within the MMS. SAXS analysis suggests potential clustering or agglomeration of MNPs, which can cause localized variations in magnetic response. These clusters may display stronger magnetic interactions or magnetization responses compared to isolated nanoparticles, contributing to variability in susceptibility measurements. Additionally, the size distribution of the MMS appears to influence this variability. Supporting Information (Figure S2C) shows the plot of magnetic susceptibility versus MMS diameter, indicating that larger microspheres exhibit reduced susceptibility, likely due to a lower concentration of iron oxide in these particles. For composite materials like chitosan microspheres containing MNPs, the magnetic susceptibility is affected by both the magnetic moments of the MNPs and their distribution within the chitosan matrix [79]. Iron oxide nanoparticles, particularly magnetite (Fe_3_O_4_) and maghemite (γ‐Fe_2_O_3_), exhibit high magnetic moments due to unpaired electrons, significantly contributing to the overall susceptibility of the composite [80]. For context, superparamagnetic polystyrene microspheres (Dynabeads) dispersed with maghemite and magnetite are reported to have susceptibilities of approximately 0.11 for M280 (12 wt% Fe) [67], 0.25 for M450 (20 wt% Fe) [81], and 0.34 for MyOne (26 wt% Fe) [67]. Compared to these values, our mean susceptibility is lower, consistent with an estimated iron content of around 1 wt%, assuming a stoichiometric and uniform distribution of MNPs within the chitosan matrix. Additionally, our susceptibility exceeds the reported 0.001 for yeast cells surface‐labeled with iron ions, which likely contain less iron [81]. Materials with positive magnetic susceptibility are classified as paramagnetic, meaning they align with rather than oppose an applied magnetic field. This positive susceptibility confirms the paramagnetic nature of our MMS [69, 82].

### 3.3 Cell Labeling with Magnetic Microspheres

**FIGURE 4:**
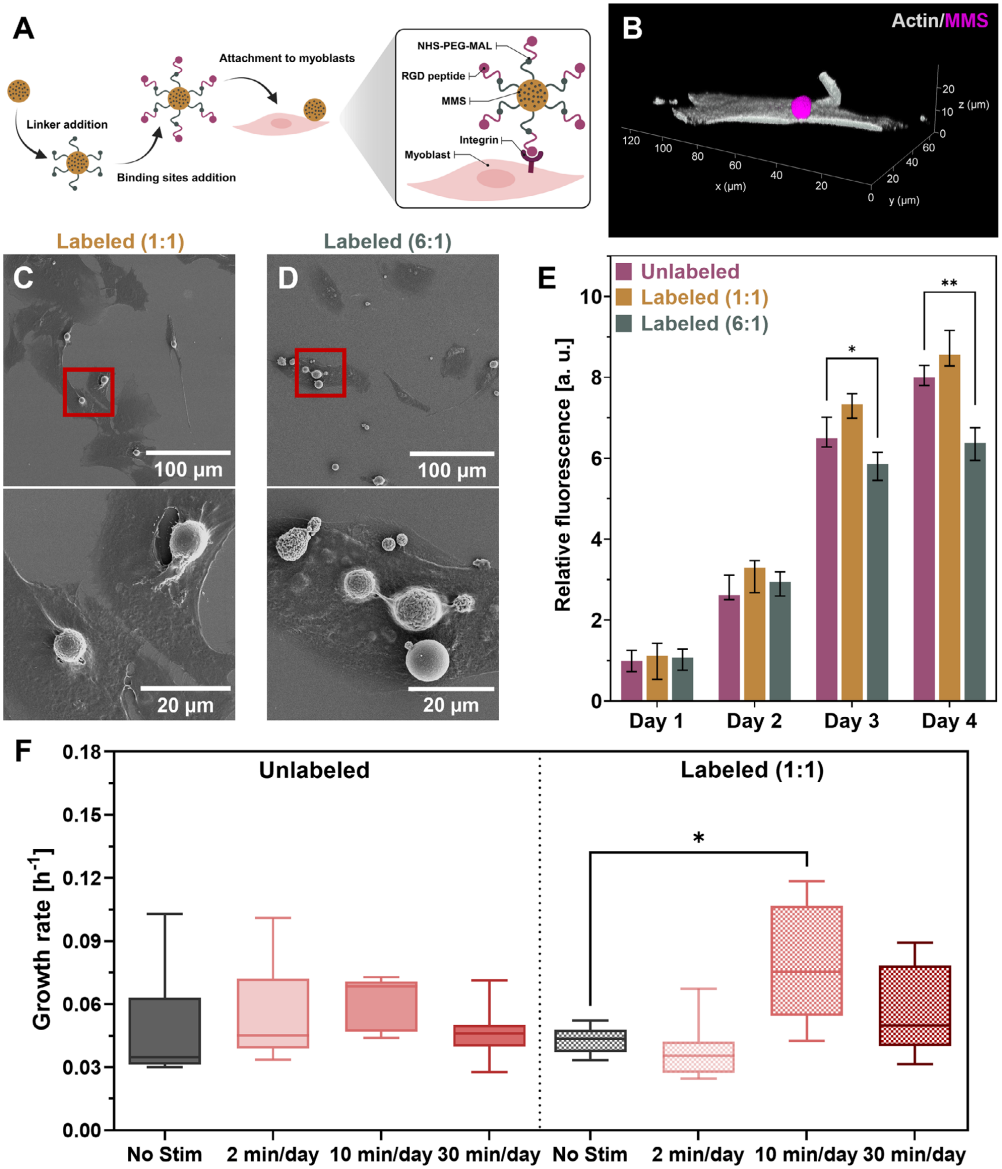
Labeled C2C12 cell behavior. (A) Schematic of MMS functionalization and of a C2C12 cell labeled with an MMS. Created in BioRender. https://BioRender.com/w81r520 (B) Confocal 3D reconstruction image applied to the z‐stack of a labeled (1:1) C2C12 cell after 24 h of culture. The color gray indicates the cell cytoskeleton stained for actin, and magenta represents the MMS from the chitosan autofluorescence. SEM images of cells labeled with a 1:1 (C) or 6:1 (D) ratio cultured on glass slides after 24 h of culture. (E) Metabolic activity of C2C12 cells labeled with a 1:1 and 6:1 ratio in comparison to unlabeled cells as a control, measured by an Alamar Blue assay over 4 days of culture. Values are median (IQR), *n* = 9 per group. Levels of statistical significance are indicated as follows: *p* < 0.05 (*), and *p* < 0.01 (**). (F) Growth rate analysis of not stimulated and 2, 10, and 30 min/day stimulated unlabeled and labeled (1:1) C2C12 myoblasts. Values were calculated from raw Alamar Blue fluorescence data by linear regression of ln(RFU) versus time during 24 and 72 h of culture. Boxplots represent the median (line), interquartile range (Q1–Q3 box), and minimum–maximum values (whiskers), *n* = 9 per group. Statistical comparisons were performed relative to the corresponding non‐stimulated control within each cell group. Levels of statistical significance are indicated as follows: *p* < 0.05 (*).

After successful production of the MMS, they were functionalized with RGD peptides to attach them to the cell membrane of the muscle cells (Figure 4A). A confocal 3D reconstruction image of labeled (1:1) cells (Figure 4B) confirmed that the MMS (magenta) is above the cell cytoskeleton (gray), showing that the MMS stays on top of the cell membranes and doesn't get taken up. This confirms the usability of the MMS size and the peptide‐functionalized MMS's successful bonding to the cell membrane without endocytosis, as represented by the schematic in Figure 4A.

To investigate the impact of the number of functionalized MMS per C2C12 cell on proliferation, different MMS‐to‐cell ratios were cultured and analyzed. After labeling by mixing the cells and MMS in suspension, the cells were seeded in well plates for 4 days, as well as on glass slides for SEM imaging. Comparison of the cell morphologies after labeling with different ratios using SEM revealed that multiple MMS per cell covered most of its surface, which might negatively influence cellular activities (Figure 4C,D). In SEM images, it can be seen that the MMS successfully remain on the membrane and are not engulfed by cells via endocytosis, adding to what was observed with confocal microscopy (Figure 4B).

The addition of foreign objects to cells can affect their viability and proliferation. Alamar Blue assay revealed that the measured fluorescence absorbance for labeled cells (1:1) was comparable to that of unlabeled cells cultured in growth medium for 4 days (Figure 4E). This means that the cells showed increased metabolic activity without being disturbed by MMS labeling. However, evaluation of a higher labeling ratio (6:1) led to significantly lower metabolic activity from day 3 onward. Based on these results, the chosen cell labeling ratio used in all subsequent experiments was 1:1.

Alamar Blue measurements were also performed over four days in both unlabeled and labeled (1:1) C2C12 cells under no stimulation and at 2 min, 10 min, and 30 min daily stimulation. Fluorescence values were normalized to the respective non‐stimulated control within each cell group to allow comparison of relative metabolic activity over time (Supporting Information, Figure S7). In both unlabeled and labeled (1:1) cells, 10 and 30 min/day stimulation resulted in a general decrease in normalized fluorescence relative to the corresponding non‐stimulated control across time points. To more directly assess proliferative behavior, growth rates were calculated from raw fluorescence data by linear regression of ln(RFU) versus time at 24 to 72 h (Figure 4F). Kinetic analysis revealed no significant change in growth rate across most conditions, except for a significant increase observed specifically in labeled (1:1) cells after 10 min daily stimulation compared to non‐stimulated labeled cells (0.075 (IQR 0.054–0.11) h^−1^ and 0.043 (IQR 0.037–0.048) h^−1^, respectively), despite the reduced normalized fluorescence observed in endpoint measurements. Notably, this interpretation is consistent with previous reports showing that electromagnetic stimulation does not adversely affect C2C12 proliferation, as assessed by colorimetric assays, despite promoting other cellular processes such as myogenic differentiation and functional signaling [83, 84]. Together, these findings demonstrate that magnetic labeling (1:1) and stimulation do not inhibit cell growth in proliferating C2C12 myoblasts.

### 3.4 Magnetic Actuation of Mechanotransduction in Labeled C2C12 Myoblasts

Cellular interactions with the surrounding microenvironment, including attachments to extracellular matrix (ECM) components and neighboring cells, are inherently mechanosensitive. For instance, stretch‐activated ion channels open when mechanical forces are applied, allowing ion fluxes that influence cellular signaling. Similarly, integrin‐associated proteins such as FAK, paxillin, talin, and vinculin are activated by mechanical load. It has been shown that integrin‐mediated signaling is involved in many cellular pathways, thereby modulating cell survival, proliferation, motility, shape, differentiation, and polarity [85, 86].

Yes‐associated protein 1 (YAP1), a mechanotransduction transcriptional regulator and component of the Hippo pathway, plays a pivotal role in mechanotransduction, translating mechanical inputs from the ECM—particularly matrix stiffness—into transcriptional responses [87]. The localization of YAP (whether in the nucleus or cytoplasm) determines its activity and is regulated by its phosphorylation state. When phosphorylated, YAP is retained in the cytoplasm in an inactive form; when dephosphorylated, it translocates to the nucleus, where it acts as a transcriptional co‐activator [88]. YAP localization has a great impact on skeletal muscle cell activity. During satellite cell maturation, YAP is predominantly localized within the nucleus of proliferating myoblasts but relocates to the cytoplasm following myogenic differentiation [87, 88].

**FIGURE 5:**
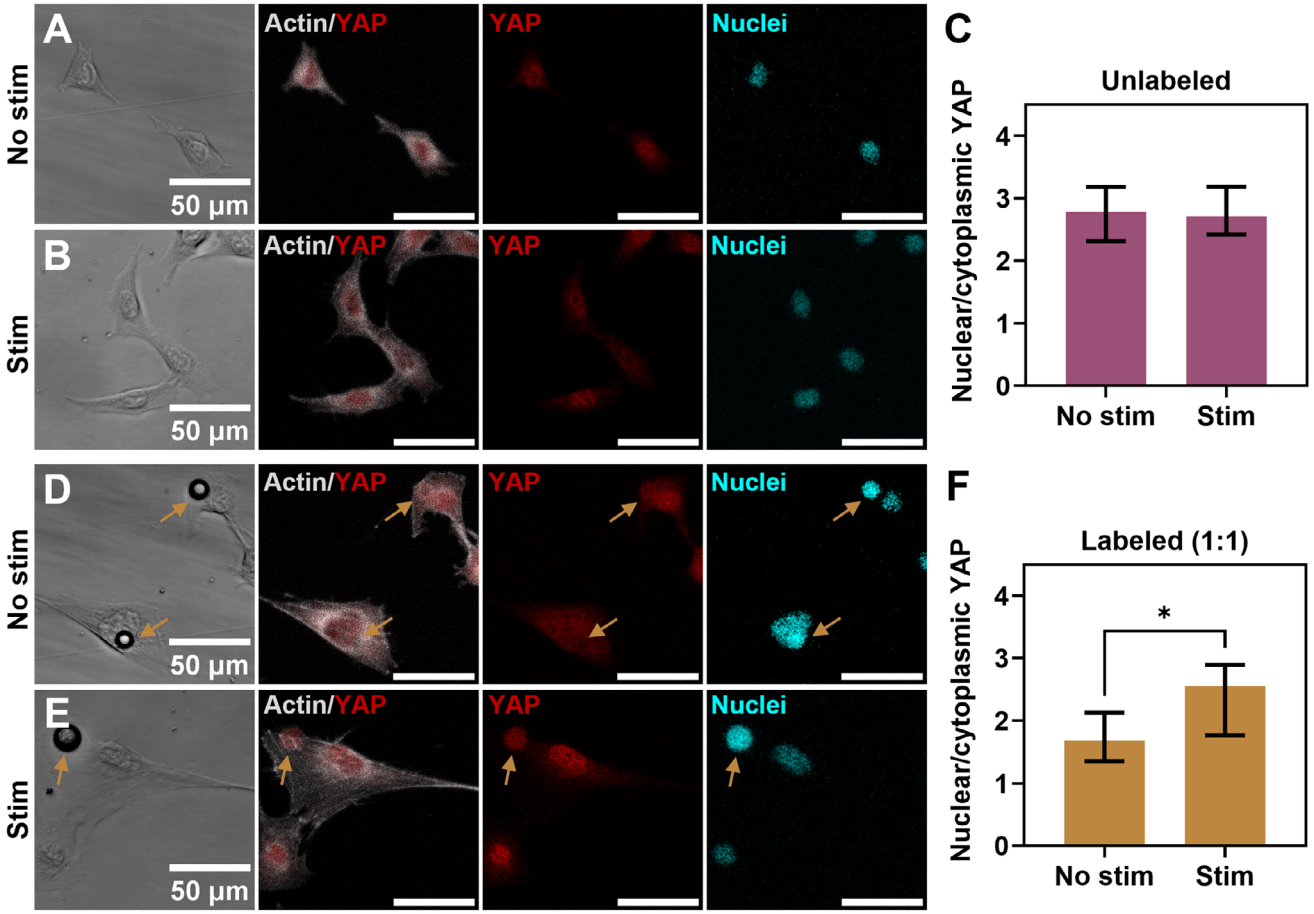
Mechanotransduction stimulation response of unlabeled and labeled (1:1) C2C12 myoblasts to dynamic magnetic stimulation. Cells were kept in growth medium for 3 days of culture and stimulated for 2 min/day with 2.9 mT at 50 Hz at RT for the last 2 days. Cells were stained for actin (cytoskeleton) with Phalloidin‐TRITC, YAP with YAP1 primary antibody and Alexa‐Fluor 488 secondary antibody, and nuclei with DAPI. On the representative fluorescent images, gray color is used for actin, red for YAP, and cyan for nuclei. Bright‐field images are shown to identify the MMS position. (A) Unlabeled C2C12 myoblast not subjected to magnetic stimulation. (B) Unlabeled C2C12 myoblast after magnetic stimulation. (C) Nuclear to cytoplasmic ratio of YAP comparison for not stimulated and stimulated unlabeled C2C12 myoblasts. (D) Labeled (1:1) C2C12 myoblast not subjected to magnetic stimulation. (E) Labeled C2C12 myoblast after magnetic stimulation. Autofluorescent MMS were identified by yellow arrows in all channels. (F) Nuclear to cytoplasmic ratio of YAP comparison for not stimulated and stimulated labeled (1:1) C2C12 myoblasts. Values are median (IQR), *n* = 15 cells per group. Levels of statistical significance are indicated as follows: *p* < 0.05 (*).

Therefore, YAP immunostaining was performed to evaluate the activation of mechanotransduction in MMS‐labeled C2C12 myoblasts exposed to dynamic magnetic stimulation during the proliferation phase. Figure 5A,B shows representative bright‐field and fluorescent images of the YAP immunostaining for unlabeled cells with no magnetic stimulation or after 2 min of magnetic stimulation for 2 consecutive days. The selection of 2 min daily magnetic stimulation is based on prior reports indicating that exposure to a 2 min dynamic magnetic field increases Ca^2+^ cytosolic concentration in C2C12 myotubes [24]. The YAP nuclear to cytoplasmic ratio (Figure 5C) didn't show a significant difference between unlabeled cells that were stimulated compared to not stimulated unlabeled cells.

Figure 5D,E shows representative bright‐field and fluorescent images of the YAP immunostaining for labeled (1:1) cells with no magnetic stimulation or after 2 min magnetic stimulation for 2 consecutive days. The YAP nuclear to cytoplasmic ratio (Figure 5F) shows a significant increase in the YAP nuclear localization on labeled (1:1) cells that were stimulated compared to not stimulated labeled (1:1) cells. The YAP nuclei‐cytoplasmic exchange is a continuous and dynamic process, controlled by post‐translational modifications, protein–protein interactions, chromatin binding, and mechanical forces that alter nuclear pore permeability. The equilibrium between nuclear import and export determines YAP distribution within the cell. Mechanical stimuli can disrupt this equilibrium, shifting YAP localization toward the nucleus and enabling its transcriptional activity [89]. In mesenchymal stem cells, it is shown that exposure to dynamic tensile loading for 30 to 360 min significantly increased the nuclear‐to‐cytoplasmic YAP ratio [86]. In human myoblasts, cyclic mechanical stretching has also been shown to promote YAP nuclear localization [90]. Therefore, the dynamic magnetic field showed no effect by itself on YAP‐mediated mechanotransduction. However, combined with MMS labeled cells, the dynamic magnetic field promotes a mechanical stimulus response in C2C12 myoblasts.

### 3.5 Dynamic Magnetic Stimulation Duration Effect on Unlabeled and Labeled C2C12 Myoblast Differentiation

**FIGURE 6:**
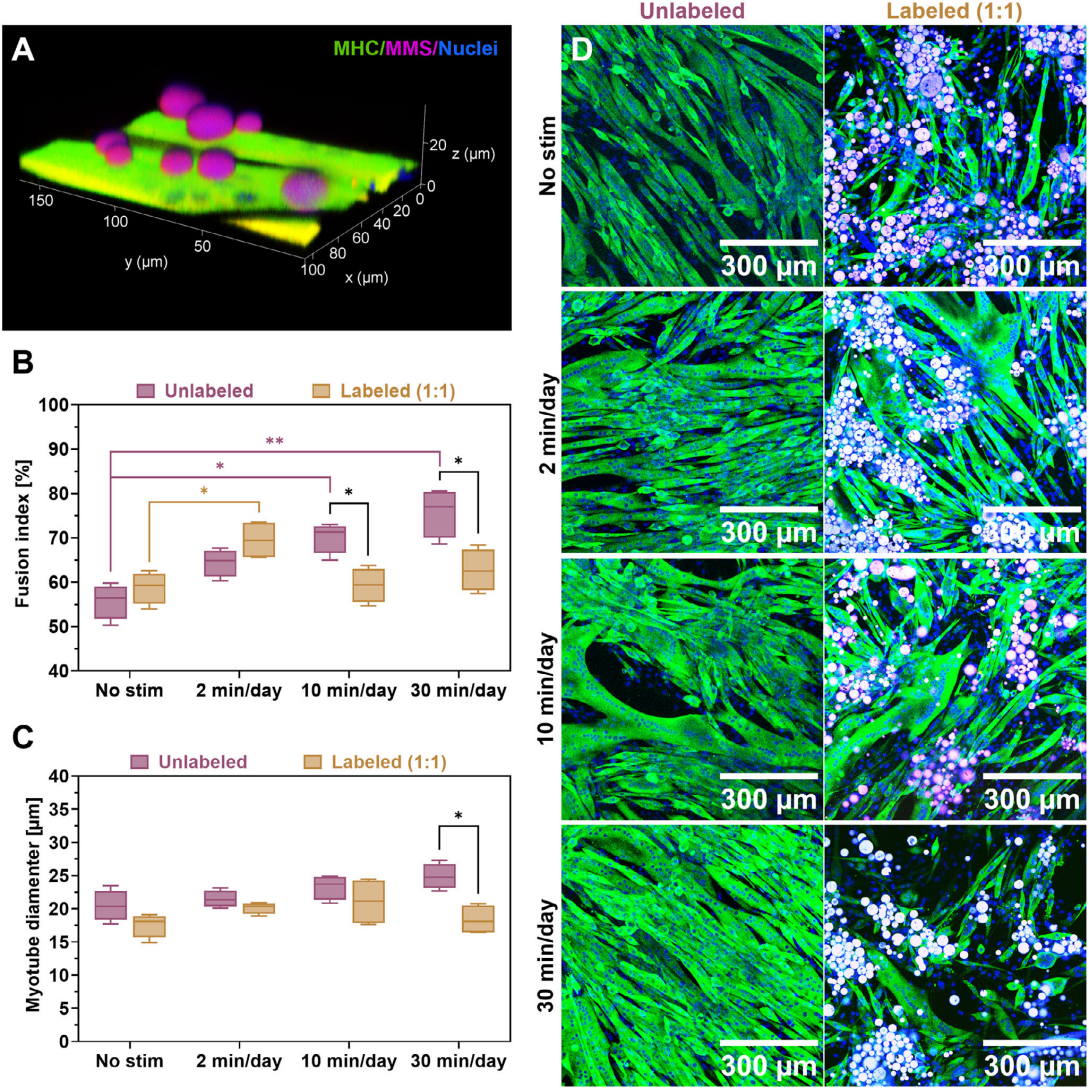
Differentiation of unlabeled and labeled (1:1) C2C12 cells using dynamic magnetic stimulation. Cells were kept in differentiation medium for 4 days of culture and stimulated for 2, 10, and 30 min/day with 2.9 mT at 50 Hz at RT. (A) Confocal 3D reconstruction image applied to the z‐stack after differentiation of labeled (1:1) C2C12. Green indicates the myosin heavy chain (MHC) from MF20 (Myh4) antibody staining, blue the nuclei from DAPI staining, and magenta represents the MMS from the chitosan autofluorescence. (B) Fusion indices of not stimulated and 2, 10, and 30 min/day stimulated unlabeled and labeled (1:1) C2C12. (C) Myotube diameters of not stimulated and 2, 10, and 30 min/day stimulated unlabeled and labeled (1:1) C2C12. (D) Merge representative confocal fluorescence images of not stimulated and 2, 10, and 30 min/day stimulated unlabeled and labeled (1:1) C2C12 myotubes stained for MHC (MF20, green) and nuclei (DAPI, blue). Autofluorescent chitosan MMS are shown in pink/purple color. Boxplots represent the median (line), interquartile range (Q1–Q3 box), and minimum–maximum values (whiskers), *n* = 4 per group. Levels of statistical significance are indicated as follows: *p* < 0.05 (*), and *p* < 0.01 (**).

The effect of dynamic magnetic stimulation duration on the myogenic differentiation of both unlabeled and labeled (1:1) C2C12 myoblasts to multinucleated myotubes was evaluated by daily exposure of C2C12 cells to a dynamic magnetic field (2.9 mT, 50 Hz). The cells were cultured for 24 h to attach and reach 95–100% confluency. From day 1 to day 5, they were stimulated once per day for 2, 10, or 30 min, while a control group was not stimulated. On day 5, the samples were stained for MHC and nuclei to show their fusion and expression of the myogenesis marker. A 3D reconstruction of a confocal fluorescent z‐stack image of labeled (1:1) cells after differentiation (Figure 6A) revealed myotube formation with bound MMS on the membrane. It shows that when multiple myoblasts with their attached MMS fuse, the MMS remain on the membrane of the newly differentiated myotubes. Additionally, spontaneous contractions of C2C12 myotubes after 4 days in differentiation medium under four conditions: not stimulated, unlabeled cells and labeled (1:1) cells, and 10 min/day magnetically stimulated, unlabeled and labeled (1:1) cells were recorded immediately after stimulation (Supporting Information Video S2). This indicates that the C2C12 myotubes' contractile behavior was sustained in the presence of the MMS.

Myotube formation was assessed by quantification of the fusion index (Figure 6B) and myotube diameter (Figure 6C) in labeled and unlabeled cells (1:1 ratio), as well as not stimulated or stimulated for various periods. Representative confocal fluorescence images of each group are shown in Figure 6D. When looking at the fusion index of unlabeled cells, stimulation for periods of 10 and 30 min/day significantly increased the index (71.4 (IQR 66.6–72.6)% and 77.1 (IQR 70.1–80.4)%, respectively) compared to no stimulation (56.4 (IQR 51.8–59.0)%). In the case of the labeled (1:1) cells, a significant increase in the fusion index was observed for 2 min/day stimulation (69.5 (IQR 65.7–73.4)%) compared to no stimulation (59.3 (IQR 55.2–61.9)%). In both 10 min/day and 30 min/day stimulation conditions, labeled (1:1) cells had a significantly lower fusion index compared to unlabeled cells at the same stimulation time condition. For no stimulation and 2 min/day stimulation, no significant difference was found between the fusion index of unlabeled and labeled (1:1) C2C12.

The median (IQR) of the myotube diameters (Figure 6C) measured in unlabeled cells that were not stimulated or stimulated for 2, 10, and 30 min/day were 20.3 (18.4–22.7) µm, 21.3 (20.4–22.7) µm, 23.7 (21.4–24.8) µm, and 24.8 (23.1–26.7) µm, respectively. These values for labeled (1:1) cells that were not stimulated or stimulated for 2, 10, and 30 min/day were 18.1 (15.7–18.9) µm, 20.3 (19.2–20.8) µm, 21.1 (17.8–24.3) µm, and 18.1 (16.4–20.5) µm, respectively. No significant difference was observed between the unlabeled and labeled (1:1) groups. However, following 30 min/day of stimulation, the mean myotube diameter was significantly lower in the labeled (1:1) group than in the unlabeled group .

**FIGURE 7:**
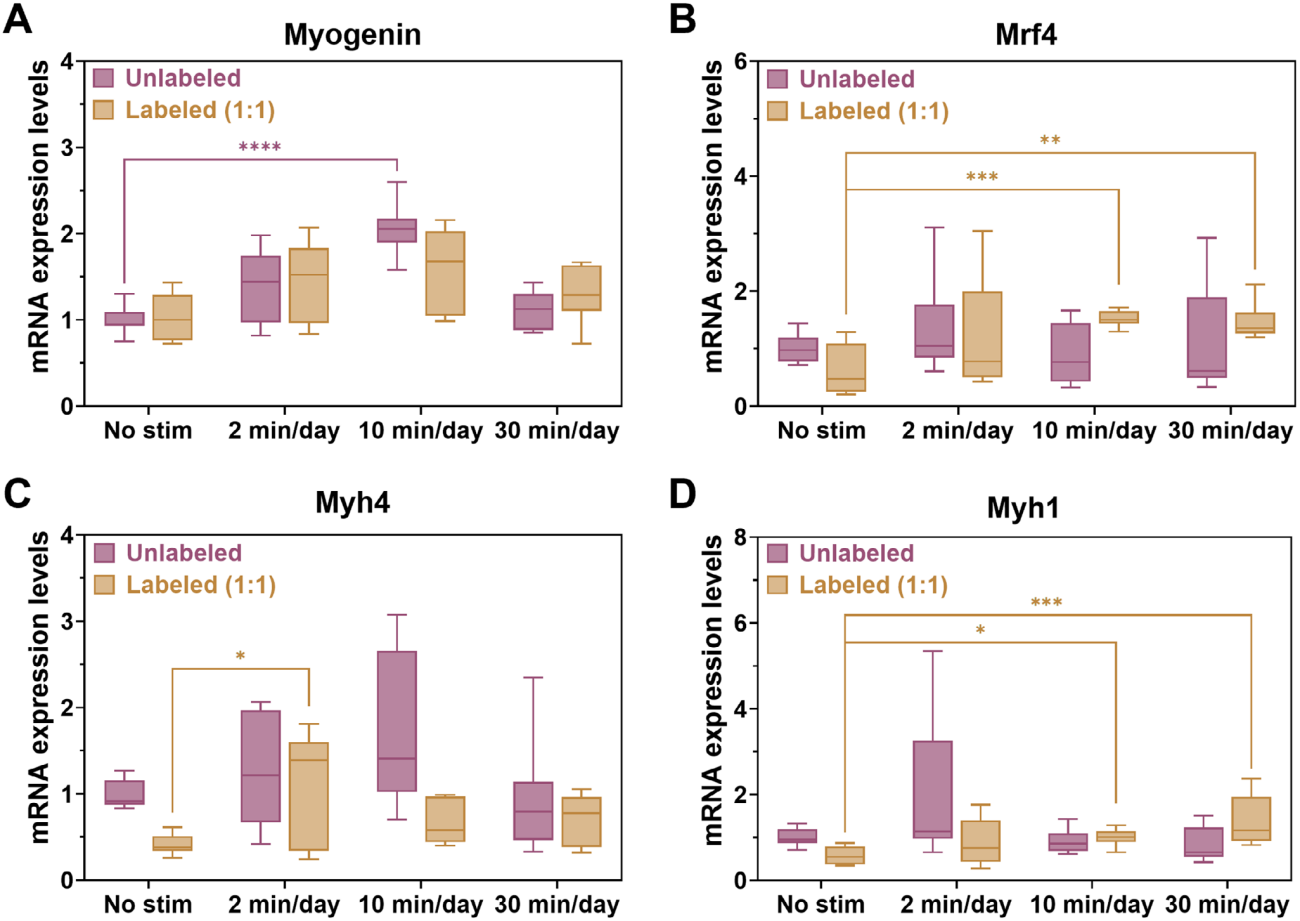
Quantification of myogenic differentiation gene expression in unlabeled and labeled (1:1) C2C12 cells after dynamic magnetic stimulation. Cells were kept in differentiation medium for 4 days of culture and stimulated for 2, 10, and 30 min/day with 2.9 mT at 50 Hz at RT. mRNA expression of not stimulated, and 2, 10, and 30 min/day stimulated unlabeled and labeled (1:1) C2C12 for (A) myogenin, (B) Mrf4, (C) Myh4, and (D) MHC‐IIdx. Boxplots represent the median (line), interquartile range (Q1–Q3 box), and minimum–maximum values (whiskers), *N* = 3 per group. Statistical comparisons were performed relative to the corresponding non‐stimulated control within each cell group. Levels of statistical significance are indicated as follows: *p* < 0.05 (*), *p* < 0.01 (**), and *p* < 0.001 (***).

In addition to the fusion index quantification, the expression levels of genes related to myotube differentiation were measured with qRT‐PCR. In agreement with the results of the fusion index, it seems that stimulation in itself (in both unlabeled and labeled cells) can affect the differentiation degree of the cells, as shown by the overall increased expression of two myogenic transcription factors (Myogenin and Mrf4) and two myosin's (Myh4 and Myh1) (Figure 7), which become upregulated in various stimulation paradigms [91, 92, 93, 94]. Although the differences between unlabeled cells exposed to various stimulation durations were not always significant, there was a clear trend toward upregulation upon stimulation. In contrast, stimulating labelled cells seemed to have a greater and often significant effect on the expression of the aforementioned genes, suggesting that myotube maturation is more advanced in labelled cells upon stimulation.

These results highlight that the dynamic magnetic field applied from our in‐house developed magnetic stimulator has a positive effect on myogenesis of C2C12 myoblasts at 10 and 30 min daily stimulation. The biological efficacy of our custom‐built system is consistent with results reported using both commercial and custom‐built Helmholtz platforms. For instance, while Sapir et al. [36, 37] utilized commercial Helmholtz coils to drive maturation in cardiac cells (5 Hz, 1–1.5 mT) and vascularization in aortic endothelial cells (40 Hz, 1–1.5 mT) through indirect stimulation in magnetic hydrogels, our platform achieves similar maturation effects in skeletal muscle cells. Furthermore, our observations of increased fusion index and upper regulation of myogenesis markers align with the findings of Terrie et al., who reported enhanced myogenesis in human muscle cells using a 75 Hz/2 mT field produced by a custom‐built setup under 2 and 22 h daily stimulation [22]. The fact that our low‐cost setup produces comparable biological outcomes to these established systems at shorter stimulation times (10 min/day) underscores its practical relevance as an accessible tool for mechanobiology research. When combined with MMS actuators, a positive effect on differentiation can already be observed at an even shorter daily stimulation time (2 min/day) by the inclusion of mechanical stimuli through the mechanotransduction response of the MMS‐cell membrane binding.

This effect is further supported by changes in MHC isoform expression, particularly the upregulation of *Myh4* (encoding MHC‐IIb) and *Myh1* (encoding MHC‐IIx) under different time conditions of stimulation. In small adult mammals, skeletal muscle expresses four main MHC isoforms (IIb, IIx, IIa, and I), which are associated with contractile properties ranging from fast glycolytic (MHC‐IIb) to slow oxidative (MHC‐I). MHC‐IIx is an intermediate phenotype, retaining fast contractile characteristics while exhibiting higher fatigue resistance than MHC‐IIb. Transitions between these isoforms follow a defined progression (IIb ↔ IIx ↔ IIa ↔ I) and are also known to be regulated by mechanical and electrical stimuli [95, 96, 97].

Previous studies in rats have shown that endurance training induces such transitions, with increased exercise duration promoting a shift from MHC‐IIb toward MHC‐IIx expression [95]. This is consistent with our findings, where short stimulation (2 min/day) primarily increased *Myh4* expression (Figure 7C), indicative of fast‐type maturation, while longer stimulation durations (10 and 30 min/day) resulted in increased *Myh1* expression in labelled (1:1) cells (Figure 7D), suggesting progression toward a more oxidative, fatigue‐resistant phenotype. Together, these results indicate that magnetic stimulation combined with MMS actuation not only enhances myogenic differentiation but also modulates fiber‐type specification in a duration‐dependent manner.

The enhancement of myogenic differentiation under magnetic stimulation is attributed to the combined electrophysiological and mechanotransduction effects of time‐varying fields. Magnetic fields interact with membrane receptors, ion channels, and cytoskeletal elements, thereby modulating intracellular signaling in a field‐dependent manner. In the low‐frequency regime, induced electric currents alter membrane activity without thermal damage, with dynamic fields being particularly effective in eliciting biological responses [20]. Exposure to extremely low‐frequency electromagnetic fields has been shown to upregulate myogenic regulatory factors and enhance gap junction formation, facilitating myotube assembly [21].

In parallel, magnetic forces acting on diamagnetic cellular components or on attached magnetic actuators provide mechanical cues that are transmitted through integrin–cytoskeleton linkages [98]. This mechanical input activates mechanotransduction pathways, such as YAP signaling, consistent with our observations. Together, the interplay between electrically induced membrane responses and cytoskeleton‐mediated mechanotransduction provides a mechanistic basis for the observed increase in myogenic differentiation under dynamic magnetic stimulation.

Approaches comparable to that described herein have been reported for other cell types like endothelial cells, tendon cells, and most prevalently bone cells. In endothelial cells, the expression of endothelin‐1 was upregulated, while in human tendon cells, targeting of integrin‐linked kinase induced phosphorylation of protein kinase B [59, 99]. For example, in bone cells, magnetic microbeads coated with RGD have previously been attached to the integrin receptors of primary human osteoblasts and stimulated with a time‐varying magnetic field (60 mT) produced by a permanent magnet. Mechanotransduction‐mediated stimulation enhanced osteoblastic activity, as evidenced by increased mineralized bone matrix formation and elevated osteopontin expression [98]. Multiple other studies have explored osteogenic differentiation by targeting integrins with RGD‐functionalized magnetic actuators [100, 101]. Magnetic stimulation with actuators has also been used to target other receptors in the cell membrane, like ion channels and growth factor receptors [41]. For instance, Rotherham et al. coated MNPs with antibodies and attached them to Wnt receptors in human mesenchymal stem cells. Subsequent magnetic stimulation with an oscillating magnetic field (25–120 mT) generated by an array of permanent magnets was shown to activate the Wnt/β‐catenin signaling pathway [102].

In summary, the integration of dynamic magnetic fields with MMS labeling presents a promising strategy to influence myoblast differentiation through remote mechanical actuation. The different responses across stimulation times underscore the complexity of magnetically driven mechanotransduction and the need for precise control over stimulation regimens. Future studies should further clarify the underlying mechanisms through broader molecular profiling and functional assays such as calcium imaging, contractility analysis, or force measurements to determine how the observed molecular and morphological changes translate into physiological performance. In addition, direct comparison with established mechanical stimulation approaches, such as cyclic stretch, would help further contextualize the mechanotransduction pathways engaged by MMS‐mediated actuation.

## 4 Conclusion

In this work, we report the production and optimization of a tunable platform for magnetic stimulation of skeletal muscle cells with a reasonable end production price. This platform allows a versatile range of magnetic stimulation of cells and eventually bioengineered tissues. A magnetic stimulator consisting of Helmholtz pair of coils can produce both static and dynamic magnetic fields with tunable magnetic flux density. For dynamic fields, the frequency and waveform can be varied depending on the attached power source.

Furthermore, we report the production of functionalized magnetic microspheres for cellular labeling via integrin receptors, which are not endocytosed by the cells, with an optimized MMS‐to‐cell ratio of 1:1. Unlabeled and labeled C2C12 cells were magnetically stimulated with a dynamic magnetic field (2.9 mT, 50 Hz) for different periods of 2, 10, and 30 min/day, which showed increased fusion indices at 10 and 30 min daily stimulation for unlabeled cells and at 2 min daily stimulation for labeled cells. Longer stimulation times of labeled cells showed a shift for the upper regulation of different myosin isoforms, consistent with exercised muscle fibers.

Taken together, these findings establish that dynamic magnetic fields and MMS labeling can be tuned to direct myogenic differentiation through mechanotransduction. Our platform provides a vast range of applications where exercising and noninvasive mechanical stimulation control over cells and bioengineered tissues is required. As control over cellular and tissue functions is also relevant for the engineering of other tissues, additional opportunities will follow from applying the platform to other cell types and extending its usage for both regenerative and bioengineering purposes.

## Conflicts of Interest

The authors declare no conflicts of interest.

## Supporting information




**Supporting File 1**: smll74473‐sup‐0001‐SuppMat.docx.

**Supporting File 2**: smll74473‐sup‐0002‐VideoS1.mp4.

**Supporting File 3**: smll74473‐sup‐0003‐VideoS2.mp4.

## Data Availability

The data that support the findings of this study are available from the corresponding author upon reasonable request.
