# Supplementary material for: A Platform for the Actuation of Magnetically Labeled Skeletal Muscle Cells Using Dynamic Magnetic Stimulation
Source: Small. 2026 Jul 14;22(49):e74473. doi: 10.1002/smll.74473 (PMC13533221; doi:10.1002/smll.74473)
Supplement: Supplementary file 1 [file SMLL-22-e74473-s002.docx]

Supporting Information

A platform for the actuation of magnetically labeled skeletal muscle cells using dynamic magnetic stimulation

Tayná C. Rodrigues^1,2,3+,^ Anna-Lena Bauknecht^1,4,5,6+^, Anna Gioran^1,2,3^, Sabine Rosenfeldt^7^, Daniel Dickes^8^, Johann Schorzmann^2,9^, Frank Döpper^2,9^, Elisabetta A. Cavalcanti-Adam^10^, Michael Maier^4^, Reinhard Richter^11^, Marissa K. Caldow^12^, Gordon S. Lynch^12^, Daniel E. Heath^4^, Andrea J. O’Connor^4^, and Sahar Salehi^1,2,3*^

^1^Chair of Biomaterials, Faculty of Engineering Science, University of Bayreuth, Bayreuth, 95447, Germany

^2^Fraunhofer Institute for Manufacturing Engineering and Automation (IPA), 70569 Stuttgart, Germany

^3^Department of Biointelligent System Engineering, Institute of Food Science and Biotechnology, University of Hohenheim, 70599, Stuttgart, Germany

^4^Department of Biomedical Engineering, Graeme Clark Institute, University of Melbourne, Victoria, 3010, Australia

^5^Empa, Swiss Federal Laboratories for Materials Science and Technology, Laboratory for Biointerfaces, Lerchenfeldstrasse 5, St. Gallen, 9014, Switzerland

^6^Empa, Swiss Federal Laboratories for Materials Science and Technology, Laboratory for Biomimetic Membranes and Textiles, Lerchenfeldstrasse 5, St. Gallen, 9014, Switzerland

^7^Chair of Physical Chemistry I, Faculty of Biology, Chemistry & Earth Sciences and Bavarian Polymer Institute, University of Bayreuth, Bayreuth, 95447, Germany

^8^Chair for Metals and Alloys, Faculty of Engineering Science, University of Bayreuth, Bayreuth, 95447, Germany

^9^Chair for Manufacturing and Remanufacturing Technology, Faculty of Engineering Science, University of Bayreuth, Bayreuth, 95447, Germany

^10^Chair of Cellular Biomechanics, Faculty of Engineering Science, University of Bayreuth, Bayreuth, 95447, Germany

^11^Experimental Physics V, Department of Physics, University of Bayreuth, Bayreuth, 95447, Germany

^12^Centre for Muscle Research, Department of Anatomy and Physiology, The University of Melbourne, Victoria, 3010, Australia

*Correspondence: Sahar Salehi, sahar.salehimueller@uni-hohenheim.de

^+^These authors contributed equally to this work.

**Table S1.** Primers and qRT-PCR conditions.

| **Gene name** | **Forward primer (concentration)** | **Reverse primer (concentration)** |
| --- | --- | --- |
| **Myogenin** | GTCCCAACCCAGGAGATCATT (100nM) | GACATATCCTCCACCGTGATG (200nM) |
| **Myh4 (MHC-IIb)** | CATCTGGTAACACAAGAGGTGC (100nM) | ATCTCAGCGTCGGAACTCAT (100nM) |
| **Myh1 (MHC-IIx)** | GAAGTTGCATCCCTAAAGGCAG (200nM) | GGAGGTAAGGAGCAGCCTC (100nM) |
| **Mrf4** | ATAACTGCTAAGGAAGGAGGAGC (200nM) | TGGAAGAAAGGCGCTGAAGA (100nM) |
| **Ywhaz** | CATTCAGCCTCGCGCTTTT  (400nM) | GATGACGTCAAACGCTTCTGG (400nM) |

qRT-PCR conditions:

Step 1: 95 °C for 120s (enzyme activation/initial denaturation)

Step 2: 95 °C for 10s (denaturation)

Step 3: 60 °C for 30s (annealing, extension, detection)

Repeat from Step 2 39 times

**Video S1.** Motion of magnetic microspheres in a 35% (v/v) glycerol/water solution toward an N45 neodymium magnet, recorded using a 10× objective in a 5 mm microchannel.

**Video S2.** Contracting C2C12 myotubes after 4 days in differentiation medium under four conditions: not stimulated, unlabeled cells and labeled (1:1) cells, and 10 min/day magnetically stimulated, unlabeled and labeled (1:1) cells. Brightfield imaging (10× objective); stimulated samples recorded immediately after stimulation.

**Figure S1:** Fabrication process of the magnetic stimulator. (A) 3D-printed circular frames for the Helmholtz coils. (B) Transformer wire was wrapped around the circular frames in the same direction on both coils. (C) The wire ends were stripped by soldering at 400 °C to create electrical contacts. (D) A lighting transformer was equipped with cable connections and a 3 A fuse for safety. (E) Computer-aided design model of the stimulator, showing the clamp (grey) that ensures the maintenance of a defined distance between the coils. (F) Picture of the complete stimulator and its connections to the lighting transformer.

**Figure S2:** Drag test to determine the magnetic susceptibility. (A) Schematic of the drag test setup. Created with BioRender.com. (B) Magnetic field distribution during the drag test. (C) Magnetic susceptibility plotted against MMS diameter.

**Figure S3:** Differentiation of C2C12 cells without (control) and with the use of PROFUSE-S1 supplement. (A) Fusion indices of control and C2C12 cells treated with PROFUSE-S1 after 3, 4, and 5 days of differentiation. (B) MHC+ area coverage of control and C2C12 cells treated with PROFUSE-S1 after 3, 4, and 5 days of differentiation. (C) Merge representative confocal fluorescence images of control and C2C12 cells treated with PROFUSE-S1 after 3, 4, and 5 days of differentiation, stained for MHC (MF20, green) and nuclei (DAPI, blue). Boxplots represent the median (line), interquartile range (Q1–Q3 box), and minimum–maximum values (whiskers), n = 3 per group. Levels of statistical significance are indicated as follows: p < 0.05 (*), p < 0.01 (**), and p < 0.001 (***).

**Figure S4:** Dimensions of the magnetic stimulator. Values are in millimeters.

**Figure S5:** Thermal images of the magnetic stimulator and transformer at different time points.

**Figure S6:** Analysis of magnetic properties. (A) Color comparison of magnetic microspheres (MMS) without (left) and with (right) magnetic nanoparticles (MNPs). (B) MMS get attracted to a permanent magnet.

**Figure S7:** Metabolic activity of not stimulated, and 2, 10, and 30 min/day stimulated unlabeled and labeled (1:1) C2C12 myoblasts, measured by an Alamar Blue assay over 4 days of culture. Values are median (IQR), n = 9 per group. Statistical comparisons were performed relative to the corresponding non-stimulated control within each cell group and time point. Levels of statistical significance are indicated as follows: p < 0.05 (*), p < 0.01 (**), and p < 0.001 (***).

**Code S1:** MATLAB Code (version R2025b) to simulate the magnetic field in the horizontal plane between the Helmholtz coils with Finite Element Method Magnetics (FEMM 4.2)

```
addpath('C:\femm42\mfiles')

%Define important variables
I = 1.22;                % Current in A
N = 200;                % Number of turns on each coil (unitless)
R = 50;                 % Radius of each coil in mm
wire_diameter = 0.6;    % Diameter of the copper wire

%Define options
Options.UseAxis = [true false false];
Options.ProblemType = 'planar';     % Can be a 2-D Planar problem ('planar') or
                                    % axisymmetric ('axi')
Options.Units = 'millimeters';      % Length units of the model
Options.Circuit = 'helmholtzcoil';  % Name of the circuit
Options.CircuitCurrent = I;         % Current in the circuit
Options.CircuitType = 1;            % 0 for parallel-connected circuit, 1 for series-
                                    % connected circuit → the specified current is
                                    % applied to each block labeled with that circuit
                                    % property. In addition, blocks that are labeled
                                    % with a series circuit property can also be
                                    % assigned a number of turns, such that the region
                                    % is treated as a stranded conductor in which the
                                    % total current is the series circuit current
                                    % times the number of turns in the region.
Options.Frequency = 50;             % Frequency of the circuit, should be 0 for a
                                    % magnetostatic problem
Options.Depth = 1;                  % Depth into the page; becomes enabled when planar
                                    % is selected
Options.Precision = 1e-8;           % Precision required for the solver, default value
                                    % is 1e-8
Options.MinAngle = 10;              % Minimum angle sent to the mesh generator

%Define materials (copper and air)
%Copper
FEMM.Material.Coil.MaterialName = 'Copper';
FEMM.Material.Coil.muX = 0.999994;              % Relative permeability μ/μ0: 0.999994
                                                % for copper wire
FEMM.Material.Coil.muY = 0.999994;              % Relative permeability μ/μ0: 0.999994
                                                % for copper wire
FEMM.Material.Coil.Hc = 0;                      % Permanent magnet coercivity: copper
                                                % wire is not a permanent magnet,
                                                % therefore 0
FEMM.Material.Coil.J = 3;                       % Real applied source current density:
                                                % 2 to 4.5 A/mm2 in copper wire
FEMM.Material.Coil.Cduct = 58;                  % Electrical conductivity of the
                                                % material: 58 Ms/m for copper wire
FEMM.Material.Coil.LamType = 3;                 % 0: Not laminated/ laminated in plane
                                                % 1: Laminated x or r
                                                % 2 → Laminated y or z
                                                % 3 → Magnet wire
                                                % 4 → Plain stranded wire
                                                % 5 → Litz wire
                                                % 6 → Square wire
FEMM.Material.Coil.nstr = 1;                    % Number of strands: 1 for Magnet wire
FEMM.Material.Coil.dwire = wire_diameter;       % Diameter of each of the wires
                                                % constituent strands in millimeters
FEMM.Material.Coil.Data = num2cell([FEMM.Material.Coil.muX, FEMM.Material.Coil.muY, ...
    FEMM.Material.Coil.Hc, FEMM.Material.Coil.J, FEMM.Material.Coil.Cduct, ...
    FEMM.Material.Coil.LamType, FEMM.Material.Coil.nstr, FEMM.Material.Coil.dwire]);
FEMM.Material.Coil.AutoMesh = 0;                % 0: mesher defers to mesh size
                                                % constraint defined in meshsize
                                                % 1: mesher automatically chooses the
                                                % mesh density
FEMM.Material.Coil.MeshSize = 5;                % Size constraint
FEMM.Material.Coil.MagDir = 0;                  % The magnetization is directed along
                                                % an angle measured in degrees
FEMM.Material.Coil.Group = 0;                   % A member of group number group
FEMM.Material.Coil.Turns = N;                   % The number of turns
Coil.Radius = R;
Coil.WindingRadius  = 7.5;

% Air
FEMM.Material.Air.MaterialName = 'Air';
FEMM.Material.Air.muX = 1;                      % Relative permeability μ/μ0: 1 for air
FEMM.Material.Air.muY = 1;                      % Relative permeability μ/μ0: 1 for air
FEMM.Material.Air.Data = num2cell([FEMM.Material.Air.muX, FEMM.Material.Air.muY]);
FEMM.Material.Air.AutoMesh = 0;                 % 0: mesher defers to mesh size
                                                % constraint defined in meshsize
                                                % 1: mesher automatically chooses the
                                                % mesh density
FEMM.Material.Air.MeshSize = 5;                 % Size constraint
FEMM.Material.Air.MagDir = 0;                   % The magnetization is directed along
                                                % an angle measured in degrees
FEMM.Material.Air.Group = 0;                    % A member of group number group
FEMM.Material.Air.Turns = 0;                    % The number of turns

% Create FEMM model
openfemm;
newdocument(0);                                 % 0: magnetics problem
                                                % 1: electrostatics problem
                                                % 2: heat flow problem
                                                % 3: current flow problem
mi_probdef(Options.Frequency,Options.Units,Options.ProblemType,Options.Precision, ...
    Options.Depth,Options.MinAngle);
mi_addcircprop(Options.Circuit,Options.CircuitCurrent,Options.CircuitType);
mi_addmaterial(FEMM.Material.Coil.MaterialName,FEMM.Material.Coil.Data{:});
mi_addmaterial(FEMM.Material.Air.MaterialName,FEMM.Material.Air.Data{:});

% Air
mi_addblocklabel(0,0);
mi_selectlabel(0,0);
mi_setblockprop(FEMM.Material.Air.MaterialName,FEMM.Material.Air.AutoMesh, ...
    FEMM.Material.Air.MeshSize,'<None>',FEMM.Material.Air.MagDir, ...
    FEMM.Material.Air.Group,FEMM.Material.Air.Turns);
mi_clearselected;

% Coils
mi_drawrectangle(-Coil.Radius(1)-Coil.WindingRadius, ...
    Coil.Radius(1)/2-Coil.WindingRadius, ...
    -Coil.Radius(1)+Coil.WindingRadius, ...
    Coil.Radius(1)/2+Coil.WindingRadius);
mi_addblocklabel(-Coil.Radius(1),Coil.Radius(1)/2);
mi_selectlabel(-Coil.Radius(1),Coil.Radius(1)/2);
mi_setblockprop(FEMM.Material.Coil.MaterialName,FEMM.Material.Coil.AutoMesh, ...
    FEMM.Material.Coil.MeshSize, Options.Circuit,FEMM.Material.Coil.MagDir, ...
    FEMM.Material.Coil.Group, FEMM.Material.Coil.Turns);
mi_clearselected;
mi_drawrectangle(-Coil.Radius(1)-Coil.WindingRadius, ...
    -Coil.Radius(1)/2-Coil.WindingRadius, ...
    -Coil.Radius(1)+Coil.WindingRadius, ...
    -Coil.Radius(1)/2+Coil.WindingRadius);
mi_addblocklabel(-Coil.Radius(1),-Coil.Radius(1)/2);
mi_selectlabel(-Coil.Radius(1),-Coil.Radius(1)/2);
mi_setblockprop(FEMM.Material.Coil.MaterialName,FEMM.Material.Coil.AutoMesh, ...
    FEMM.Material.Coil.MeshSize, Options.Circuit,FEMM.Material.Coil.MagDir, ...
    FEMM.Material.Coil.Group,FEMM.Material.Coil.Turns);
mi_clearselected;
mi_drawrectangle(Coil.Radius(1)-Coil.WindingRadius, ...
    Coil.Radius(1)/2-Coil.WindingRadius, ...
    Coil.Radius(1)+Coil.WindingRadius, ...
    Coil.Radius(1)/2+Coil.WindingRadius);
mi_addblocklabel(Coil.Radius(1),Coil.Radius(1)/2);
mi_selectlabel(Coil.Radius(1),Coil.Radius(1)/2);
mi_setblockprop(FEMM.Material.Coil.MaterialName,FEMM.Material.Coil.AutoMesh, ...
    FEMM.Material.Coil.MeshSize, Options.Circuit,FEMM.Material.Coil.MagDir, ...
    FEMM.Material.Coil.Group,-FEMM.Material.Coil.Turns);
mi_clearselected;
mi_drawrectangle(Coil.Radius(1)-Coil.WindingRadius, ...
    -Coil.Radius(1)/2-Coil.WindingRadius, ...
    Coil.Radius(1)+Coil.WindingRadius, ...
    -Coil.Radius(1)/2+Coil.WindingRadius);
mi_addblocklabel(Coil.Radius(1),-Coil.Radius(1)/2);
mi_selectlabel(Coil.Radius(1),-Coil.Radius(1)/2);
mi_setblockprop(FEMM.Material.Coil.MaterialName,FEMM.Material.Coil.AutoMesh, ...
    FEMM.Material.Coil.MeshSize, Options.Circuit,FEMM.Material.Coil.MagDir, ...
    FEMM.Material.Coil.Group,-FEMM.Material.Coil.Turns);
mi_clearselected;
mi_makeABC();

mi_addnode(-15,-15);
mi_addnode(-15,15);
mi_addnode(15,-15);
mi_addnode(15,15);
mi_addnode(0,1);

%Save the file temporarily
mi_saveas('C:\femm42\temp1.fem');        % Change this to the desired path

%Analysis
mi_createmesh();
mi_analyze;
mi_loadsolution;
mo_showdensityplot(1,0,1e-2,1e-4,'bmag');
```

*Published with MATLAB® R2025b*
